# Metabolic modelling uncovers the complex interplay between fungal probiotics, poultry microbiomes, and diet

**DOI:** 10.1186/s40168-024-01970-2

**Published:** 2024-12-20

**Authors:** Montazar Al-Nijir, Christopher J. Chuck, Michael R. Bedford, Daniel A. Henk

**Affiliations:** 1 Department of Chemical Engineering, University of Bath, Bath, BA2 7AY UK; 2 Department of Life Sciences, University of Bath, Bath, BA2 7AY UK; 3 AB Vista, Blenheim Rd. Marlborough, Woodstock Court, UK

## Abstract

**Background:**

The search for alternatives to antibiotic growth promoters in poultry production has increased interest in probiotics. However, the complexity of the interactions between probiotics, gut microbiome, and the host hinders the development of effective probiotic interventions. This study explores metabolic modelling to examine the possibility of designing informed probiotic interventions within poultry production.

**Results:**

Genomic metabolic models of fungi were generated and simulated in the context of poultry gut microbial communities. The modelling approach correlated with short-chain fatty acid production, particularly in the caecum. Introducing fungi to poultry microbiomes resulted in strain-specific and diet-dependent effects on the gut microbiome. The impact of fungal probiotics on microbiome diversity and pathogen inhibition varied depending on the specific strain, resident microbiome composition, and host diet. This context-dependency highlights the need for tailored probiotic interventions that consider the unique characteristics of each poultry production environment.

**Conclusions:**

This study demonstrates the potential of metabolic modelling to elucidate the complex interactions between probiotics, the gut microbiome, and diet in poultry. While the effects of specific fungal strains were found to be context-dependent, the approach itself provides a valuable tool for designing targeted probiotic interventions. By considering the specific characteristics of the host microbiome and dietary factors, this methodology could guide the deployment of effective probiotics in poultry production. However, the current work relies on computational predictions, and further in vivo validation studies are needed to confirm the efficacy of the identified probiotic candidates. Nonetheless, this study represents a significant step in using metabolic models to inform probiotic interventions in the poultry industry.

Video Abstract

**Supplementary Information:**

The online version contains supplementary material available at 10.1186/s40168-024-01970-2.

## Introduction

Since an EU ban in 2006, antibiotic growth promoters (AGPs) [1], once hailed as agriculture’s miracle boosters, have faced increasing scrutiny. However, rapidly escalating misuse fuelled concerns, including illegal antibiotic additions to improve productivity and prevent food spoilage [2]. Decades of antibiotic overuse in both humans and animals have resulted in the rise of antimicrobial resistance, resulting in the death of 75,000 people annually. This figure will likely only grow [3], forcing us to look for alternatives and consider a ‘one health strategy’. Yet, despite the EU ban in 2006 globally and voluntary reductions in use in the USA, 65% of antibiotics in the USA are still used in food animals [4], increasing legislative pressure to deliver ever-tightening restrictions such as bans on antimicrobials like zinc oxide and routine therapeutic antibiotic use in the EU [5, 6], creating a pressing need for alternatives.

Probiotics have emerged as a promising solution to this challenge. In-feed probiotics improve health markers and feed intake (FI), feed conversion ratio, and reduced mortality [7–10]. Additionally, they positively impact meat quality through increased protein content and a more favourable amino acid profile [10, 11]. These beneficial effects are believed to be mediated through the modulation of the gut microbiome, immune system, intestinal pH, inhibition and enzymatic activity, and various other methods of action [12].

Ilya Ilyich Mechnikov first coined the term ‘probiotic’ after observing improved health and longevity in individuals who regularly consumed yoghurt [13]. This discovery marked the beginning of our understanding of the practice dating back millennia [14], where the use of beneficial microorganisms for health had been inherently present in fermented milk products [15]. Historically, probiotics such as *Bifidobacterial* and *Lactobacilli* have been integral in human and animal health, a testament to the long-standing relationship between humans and these organisms. Despite this rich history, the sourcing and selection of probiotics have relied on traditional methods, such as fermented foods or isolating strains from hosts [16].

In the standard approaches, isolated strains undergo basic in vitro assessments for survivability, adherence, antimicrobial capacity, and lack of toxicity [17, 18]. However, a significant drawback persists: these methods offer limited insight into the probiotic’s potential effect within the complex ecosystem of the host’s gut microbiome. This lack of predictive power can lead to inconsistent or suboptimal outcomes. For instance, De Waard et al. [19] demonstrated that the composition of indigenous *Lactobacillus* populations in rats and mice was influenced more by environmental factors, such as the animal facility, than host genetics. Furthermore, Zmora et al. (2018) [20] found person-, strain-, and region-specific colonisation resistance to probiotics in humans, displaying the complexity of probiotic-host interactions. The authors suggest that this marked and person-specific mucosal colonisation resistance may explain the high variability in probiotic effects noted in previous works. This variability in the gut microbiome across different environments highlights the limitations of traditional probiotic sourcing methods in predicting the efficacy of probiotics in various hosts and contexts.

To address this knowledge gap, we propose using community metabolic modelling, which represents a transformational approach to overcome these limitations. Genome-scale metabolic models and classical FBA (flux balance analysis) have shown increasing popularity in industrial applications where metabolic models inform on how to improve productivity and elucidate key metabolic differences between species [21].

Metabolic models are mathematical representations of an organism’s metabolic pathways, constructed from annotated genomes and known enzymatic reactions. Flux balance analysis (FBA) is the most used method to study these models. FBA represents the metabolic model as a stoichiometric matrix. The matrix is constrained by enzymatic capacity and nutrient availability. FBA assumes a steady state (the sum of fluxes producing a metabolite must equal the sum of all fluxes consuming that metabolite). Given an objective function (e.g. growth rate), FBA optimises the flux distribution through the network to maximise or minimise the objective while satisfying the constraints [22, 23]. Community modelling tools extend this further to optimise the growth objective within complex microbiomes. MiCOM takes an input of genome-scale models for individual species and a diet representation. It then uses a two-step ‘cooperative trade-off’ approach to simulate the growth and metabolic interactions. First, the community growth rate is maximised using FBA, then a trade-off is set between 0 and 1 to constrain the community growth rate while minimising the regularisation term (sum of squared growth rates, which distributes growth across all species) distributing growth across all individuals in the community. This results in a solution where the individual growth rate is maximised without diminishing the growth of the other species within the community. The output of this is the relative growth rate and metabolic fluxes of each species in the community [24]. This approach allows for perturbations, such as introducing a probiotic to the community to be tested and how this would impact the microbiome. These models can predict critical outcomes such as probiotic growth within the community and outputs and inputs by predicting growth, metabolite production, consumption rates, and overall metabolic capacity.

This offers a novel, systems-level perspective that goes beyond the traditional probiotic application methods by providing a comprehensive understanding of the complex interactions between probiotics and the host microbiome. It enables a data-driven assessment of microbe-microbe interactions within the complex gut environment, predicting probiotic performance and, significantly, revealing less obvious probiotic candidates. Furthermore, leveraging resources such as the CarveFungi dataset of fungal metabolic models, this approach highlights the potential of underutilised probiotic fungi [25]. Although the success of metabolic modelling in analysing the human gut microbiome highlights its potential [26], there are currently few applications of metabolic models used in agriculture. Among the most promising studies so far are those in aquaculture, where researchers have applied similar approaches to investigate the effects of novel feed ingredients on the gut microbiota of Atlantic salmon. These studies used metagenomic data and genome-scale metabolic models to show that different yeast species [27] and black soldier fly larvae meals [28] can differentially modulate the composition and predict the metabolic capacity of the salmon gut microbiota. For livestock, the poultry industry offers an even more significant potential advantage of using metabolic modelling with controlled diets and management practices, leading to more defined modelling parameters. This combination of control and metabolic modelling could allow for precise-strain-level probiotic prescriptions.

While yeast-based probiotics have been used since the 1990s to improve growth and feed efficiency [27–30], only a few species, like Saccharomyces, have been thoroughly studied for their probiotic potential [31]. Novel probiotic species, such as *Meyerozyma guilliermondii *[32]* Chrysonilia crassa *[33, 34] and *Metschnikowia pulcherrima* [35] have recently shown promise but require further investigation. A deeper understanding of how fungi can modulate the poultry gut microbiome and the ability to explore the full biodiversity of the fungi kingdom is required to develop tailored probiotic and prebiotic formulations.

We hypothesise that metabolic modelling provides a valuable tool, beyond traditional species-level classification, for understanding probiotic interactions in the poultry gut. While limited by current poultry metagenomic datasets, our study paves the way for future developments leading to customised probiotic interventions. By leveraging metabolic modelling to identify promising underutilised fungi and focusing on their impact on health outcomes, we aim to contribute to developing effective alternatives to AGPs and improve animal health in the poultry industry.

## Materials and methods

### CarveFungi

We employed community metabolic interaction modelling to investigate the interactions between microbes within the complex gut microbiome. This data-driven approach enables a detailed analysis of how different microorganisms metabolically influence each other within a set environment. CarveFungi [25], a specialised tool for fungi, streamlines this process by creating genome-scale metabolic models (GEMs). CarveFungi utilises deep learning, drawing upon extensive metabolic databases to construct compartmentalised, fungi-specific metabolic models. This offers a distinct advantage to manual model creation, which can be more time-consuming and less tailored to fungal characteristics.

To demonstrate this, we produced a metabolic model for the *Metschnikowia pulcherrima* strain ICS1, selected for its probiotic properties [35] to initiate CarveFungi. We provided the following inputs:

- Annotated genome of *M. pulcherrima*ICS1: includes genome sequencing data and functional annotations obtained using WebAugustus [36]
- Universal fungal template: provided by CarveFungi, this integrates a comprehensive database of core fungal metabolism.

**Fig. 1:**
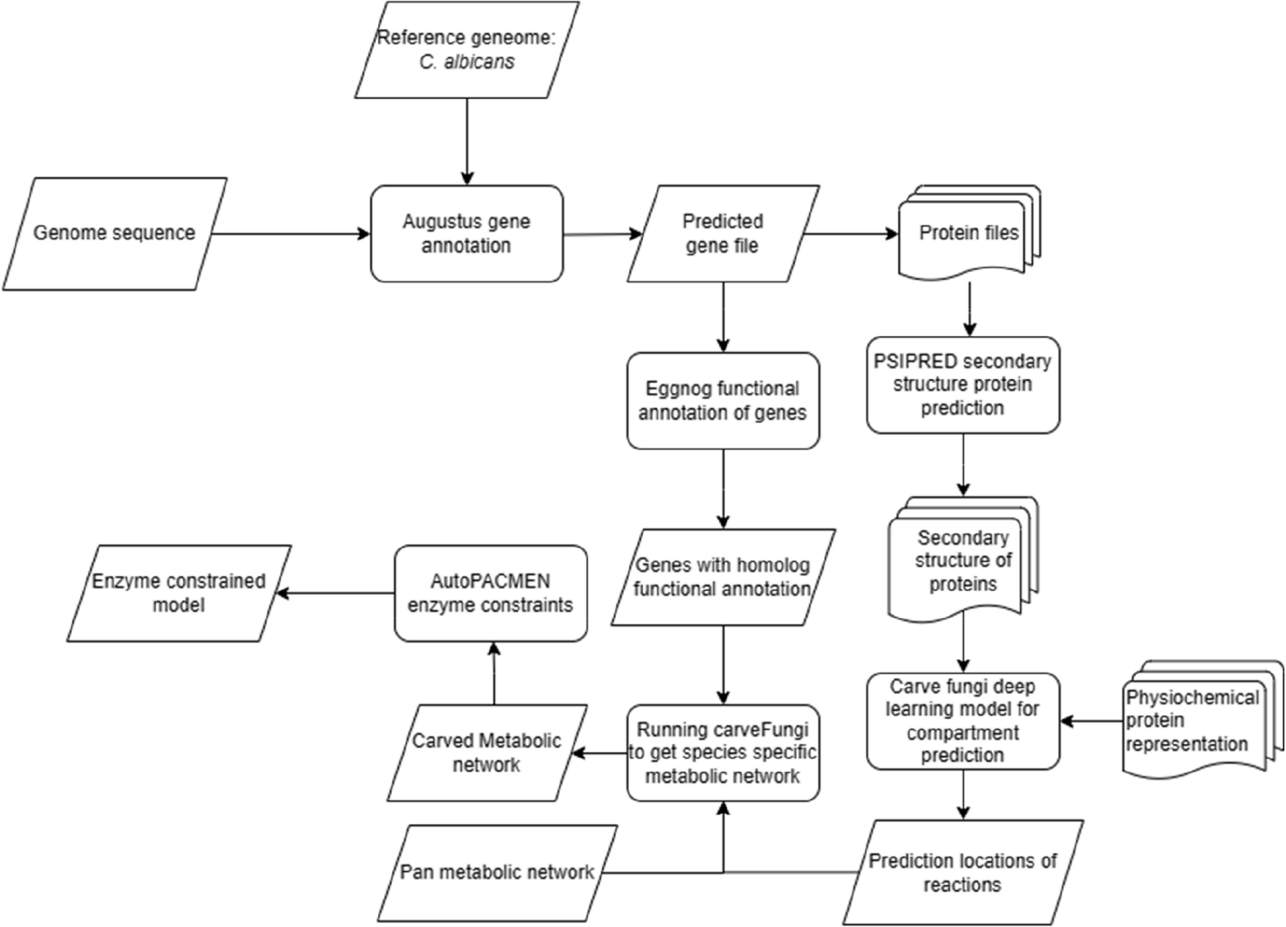
CarveFungi workflow for *Metschnikowiapulcherrima* ICS1 model generation. Input preparation includes gene prediction (WebAugustus), functional characterisation (EggNOG-mapper), and secondary protein prediction (PSIPRED)—details presented in CarveFungi toolbox

CarveFungi’s primary output for this study was a detailed GEM for *M. pulcherrima*, which was used for subsequent simulations and community analyses (Fig. 1).

Methods Toolbox: CarveFungi input preparation:

- Gene prediction: Web Augustus (reference model *Candida Albicans* for *M. pulcherrima*) (version 3.3.3).
- Functional characterisation: EggNog-Mapper (Version 5) (against diamond database) [37, 38].
- Secondary protein production: PSIPRED (Version 4) [39].

### AutoPACMEN for enzymatic constraint integration in genome-scale models

We integrated enzyme constraints into the SBML metabolic models generated by CarveFungi [25], utilising AutoPACMEN [40] to enhance metabolic simulation accuracy by accounting for enzyme capacity. AutoPACMEN leverages comprehensive databases, such as Braunschweig Enzyme Database (BRENDA) [41], Biochemically, Genetically and Genomically structured genome-scale metabolic network reconstruction database (BIGG) [42] and System for the Analysis of Biochemical Pathways-Reaction Kinetics database (Sabio-RK) [43] to establish gene-enzyme-reaction associations. Protein molecular weights were primarily from the Uniprot Database [44], with Uniparc [44] as the alternative source where UniProt data was unavailable. Due to limited fungi-specific experimental data, AutoPACMEN’s default protein concentration (0.095 g/dcw) was employed, enabling robust enzymatically constrained models.

### MiCOM

MiCOM [24] was selected as our modelling platform due to its established strengths in traceability, reproducibility, and comprehensive documentation [45]. Its success in simulating human gut microbiomes [46–48], including SFCA prediction and disease model analysis, further support its suitability for this study. We employed MiCOM to investigate metabolic interactions within the broiler gut microbiome, specifically focusing on fungal influence. Leveraging the AGORA (assembly of gut organisms through reconstruction and analysis) database [49], a comprehensive resource of gut bacteria metabolic models readily applicable to monogastric communities, we analysed the metabolic influence of fungi on microbial communities. Specifically, we examined their impact on how fungi can modulate a microbiome and the potential inhibition of problematic pathogens like *Salmonella*, *Shigella*, and *Clostridium* [49–52].

To ensure a comprehensive bacterial community representation, a relative abundance of 0.0001 was applied. Our simulations utilised a cooperative trade-off value of 0.7 (unless otherwise stated), as this setting optimally balanced individual species growth within the overall community structure.

#### Simulation of disease models

To investigate the impact of fungi on pathogenic bacteria, we conducted simulations for each metagenomic sample. Within each simulation, we introduced a target pathogen (*Salmonella*, *Shigella* or *Clostridium*) at an inclusion of 0.1. Simultaneously, a single probiotic fungus candidate was added at an inclusion level of 0.05. Simulations were based on metagenomic samples from Liao et al. [53]. Our study from this point focused on 90 fungal strains selected based on the available literature, which showed non-toxicity (see Supplementary Table S1 for a complete list) [35, 54–135].

#### Construction of the diets

Two diets were used, one of which was a corn/soybean meal diet representative of a finisher diet [136] and the other was obtained from Liao et al. [53] as an average between starter and finisher diets. Ileal digestibility was estimated based on available digestibility literature [137, 138]. Both were used to determine diet sensitivity during the simulations and to validate SFCA production. A detailed breakdown of digestibility calculations can be found in the supplementary (Supplementary Table S2) [137, 139–141].

Estimation of nutrient flux available to gut microbiota post-ileal digestion reaching the gut microbiota after digestion. Factoring in ileal digestibility, component mass, molecular weight (MW), diet composition and consumption.

1 $${\text{Flux }\left(\text{mMg}^{-1}\text{h}^{-1}\right)}_\text{component}=\frac{\sum\left(1-{\text{Ileal digestibilty}}_\text{Component}\right)\times{\text{relative abundance }(\text{g}^{-1})}_\text{component}\times\text{grams eaten }\left(\text{g}^{-\text{h}}\right)\times1000}{{\text{MW}}_\text{component}}$$

**Fig. 2:**
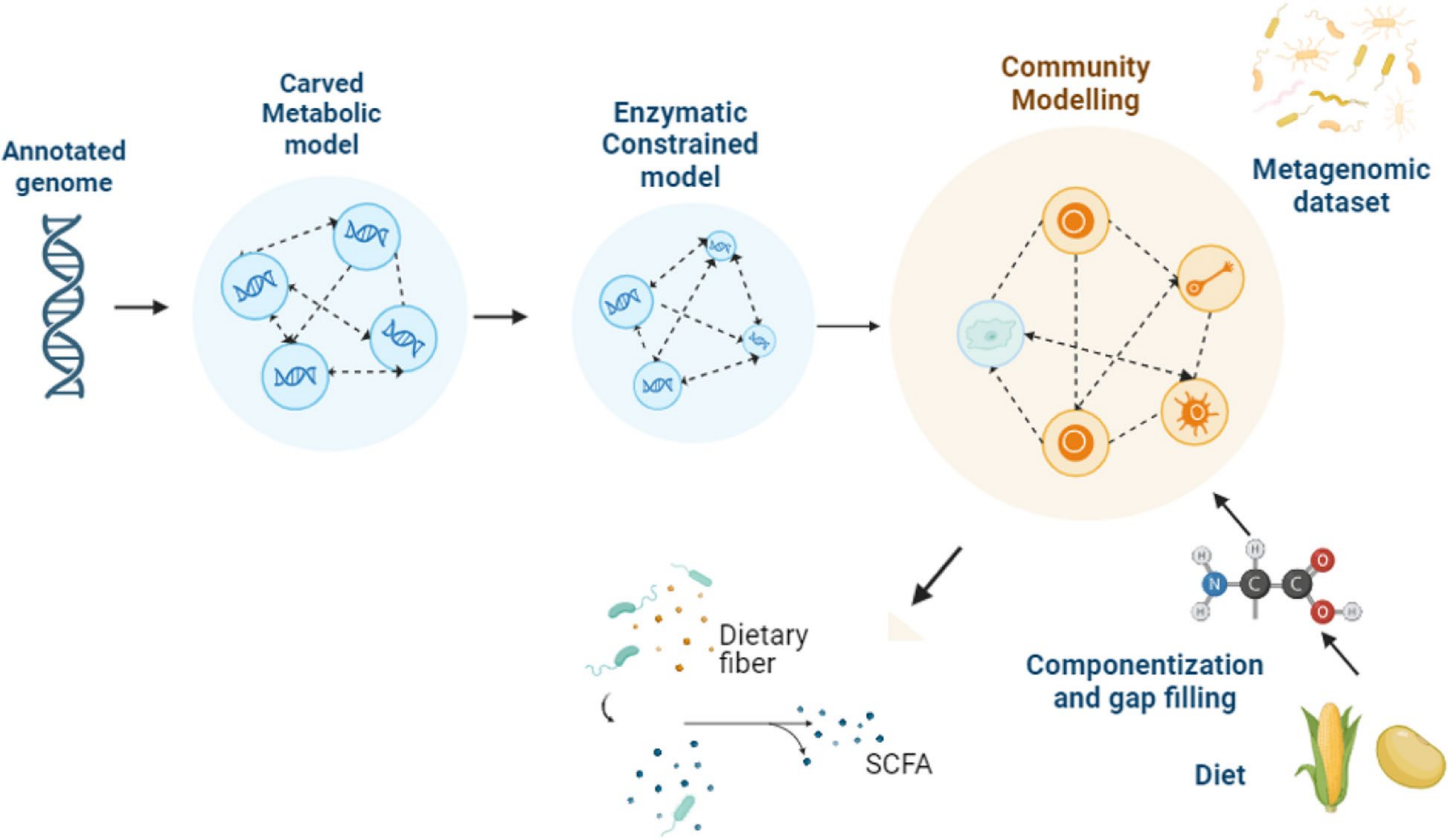
Computational workflow. Metabolic models generated by CarveFungi and enhanced with enzyme constraints (AutoPACMEN) are used for community simulations within MiCOM. This workflow enables the analysis of microbial interactions and metabolic output within the gut environment. Created in BioRender. Al-nijir, M. (2024) https://BioRender.com/d32b199

Bile acids were added according to literature values [140]. Finally, gap-filling was performed against a manifest of broiler-associated microbes [142]. This manifest was limited to microbes found within AGORA to ensure compatibility with the AGORA database (Fig. 2).

### Data extraction and analytical tools

For data extraction, WebPlotDigitizer (version 4.7) [143] was employed to digitize data from the study by Liao et al. [53].

Subsequent data analysis and visualization were performed using Python (version 3.8) [144]. The pandas library (version 1.5.3) facilitated data manipulation [145], while Maplotlib (version 3.7.3) [146] and Seaborn (version 0.12.2) [147] were used for creating graphs.

Additionally, OpenAI’s GPT-4 was instrumental in code generation and debugging [148]. All code used to run modeling and do the analysis is available on: Montazar1234/PoultryProbioticModels (github.com).

Weighted Mtype Score calculation, where the score for each genus is multiplied by its abundance and growth rate (μ) to assess the cumulative impact of fungi on the bacterial community

2 $$\text{Weighted Mtype Score}={\text{Abundance}}_{\text{genus}} \times\upmu \times {\text{Mtype Score}}_{\text{genus }}$$

Shannon diversity index was used to quantify the diversity of organisms within the simulated microbial communities (Eq. 3). Microbiomes with high diversity are considered more robust and resilient, offering protection against colonization by novel organisms.

Shannon index, where H is the Shannon diversity index, a measure of a community's diversity, Pi is the proportion of individuals that belong to that species, and S is the total number of species. This index measures richness (the number of distinct species) and evenness (the relative abundance of each species).

3 $$H=-\sum\limits_{i=1}^{s}{p}_{i}\text{ln}({p}_{i})$$

To infer potential shifts in community diversity, the Shannon index was recalculated to include alterations in abundance as a function of change in growth rate due to the introduction of fungi. Growth rates were normalised to proportions using Eq. 4.

Equation for normalising, growth rates to proportions: *G*_i_ represents the growth rate of the *i*th microbe, and n is the total number of microbes within the sample. *G*_j_ is the sum of the growth rate of all microbes within a sample.

4 $${p}_{i}=\frac{{G}_{i}}{{\sum }_{j=1}^{n}{G}_{j}}$$

## Results

### Validation Of SFCAs

To validate our metabolic modelling approach, we compared predicted SCFA production fluxes with measured SCFA concentrations from the study by Liao et al. [53], which reported metagenomic profiles, diets, and SCFA concentrations over time in different gut sections of broilers. Simulations were performed using an approximation of the reported diet (Diet 2) and using two trade-off values, 0.7 (A) and 0.8 (B).

Pearson correlation coefficients between simulated and measured SCFA levels varied across gut sections and simulation parameters (Table 2). Several SCFAs showed statistically significant positive correlations, particularly in the caecum. Diet 2 and a 0.7 trade-off, propionate, isobutyrate, butyrate in the caecum, and acetate in the duodenum, all had Pearson values > 0.95 (*p* < 0.05). Propionate in the caecum was consistently predicted across all tested perturbations, with significant correlations in all cases.

**Fig. 3:**
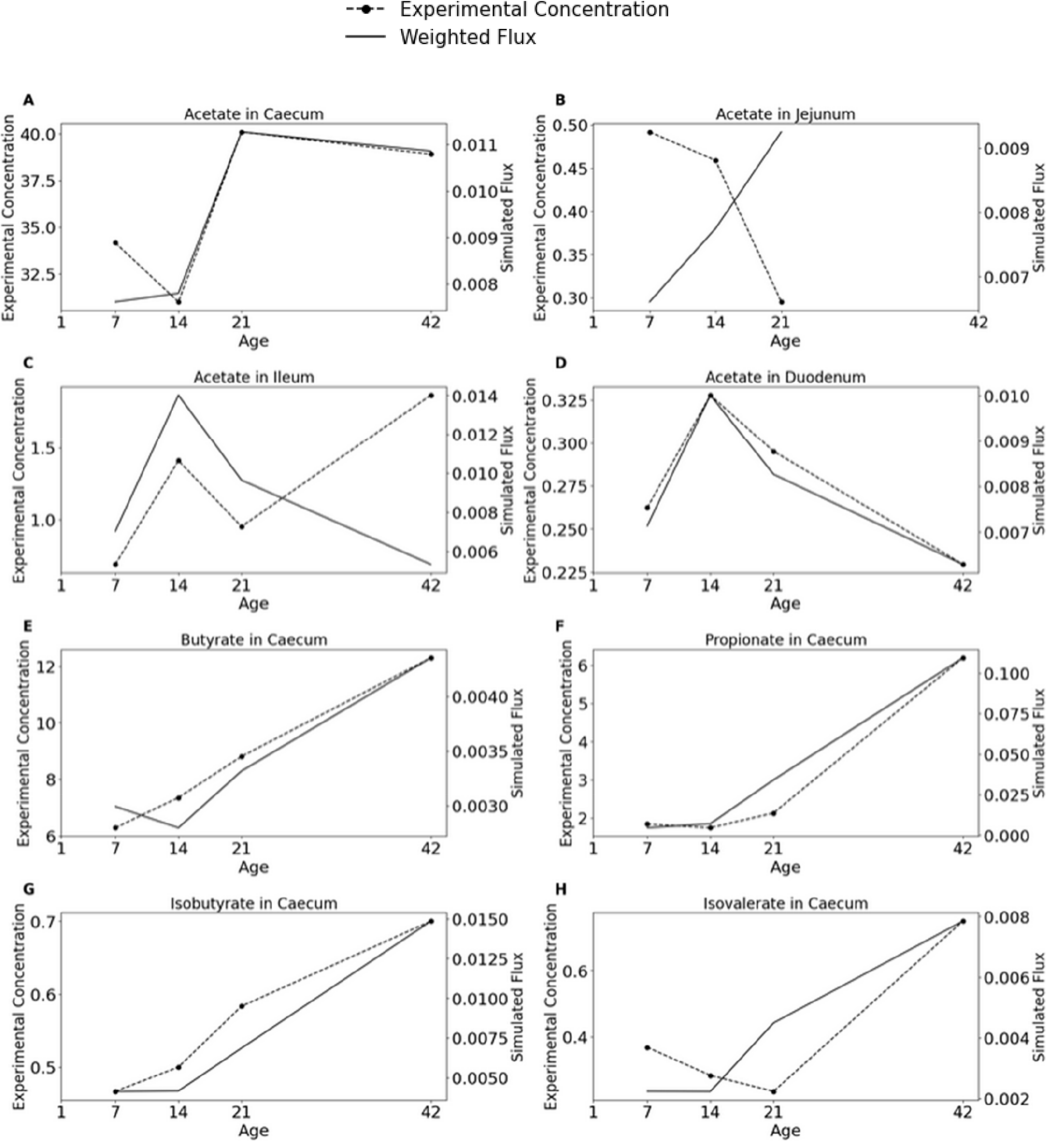
Comparative analysis of experimental SCFA concentrations and predicted weighted fluxes in gut sections over time

To visualise the agreement between simulated and measured SCFA production, we compared temporal concentrations of all measured and predicted flux SCFAs across different gut sections (Fig. 3). Despite each time point being simulated independently, the predicted fluxes followed similar patterns as the observed concentration.

### Introduction of fungi to microbial communities

Fungal strains were introduced at a relative abundance of 0.05 into simulated microbial communities derived from a metagenomic dataset [142]. That included duodenal, caecal, and faecal microbiota at 1, 7, 21, and 35 days of age. Simulators were run for fungal genomic metabolic models that matched the AGORA database at above 50% abundance and were reported to be in broilers [195].

#### Positive and negative interactions

**Fig. 4:**
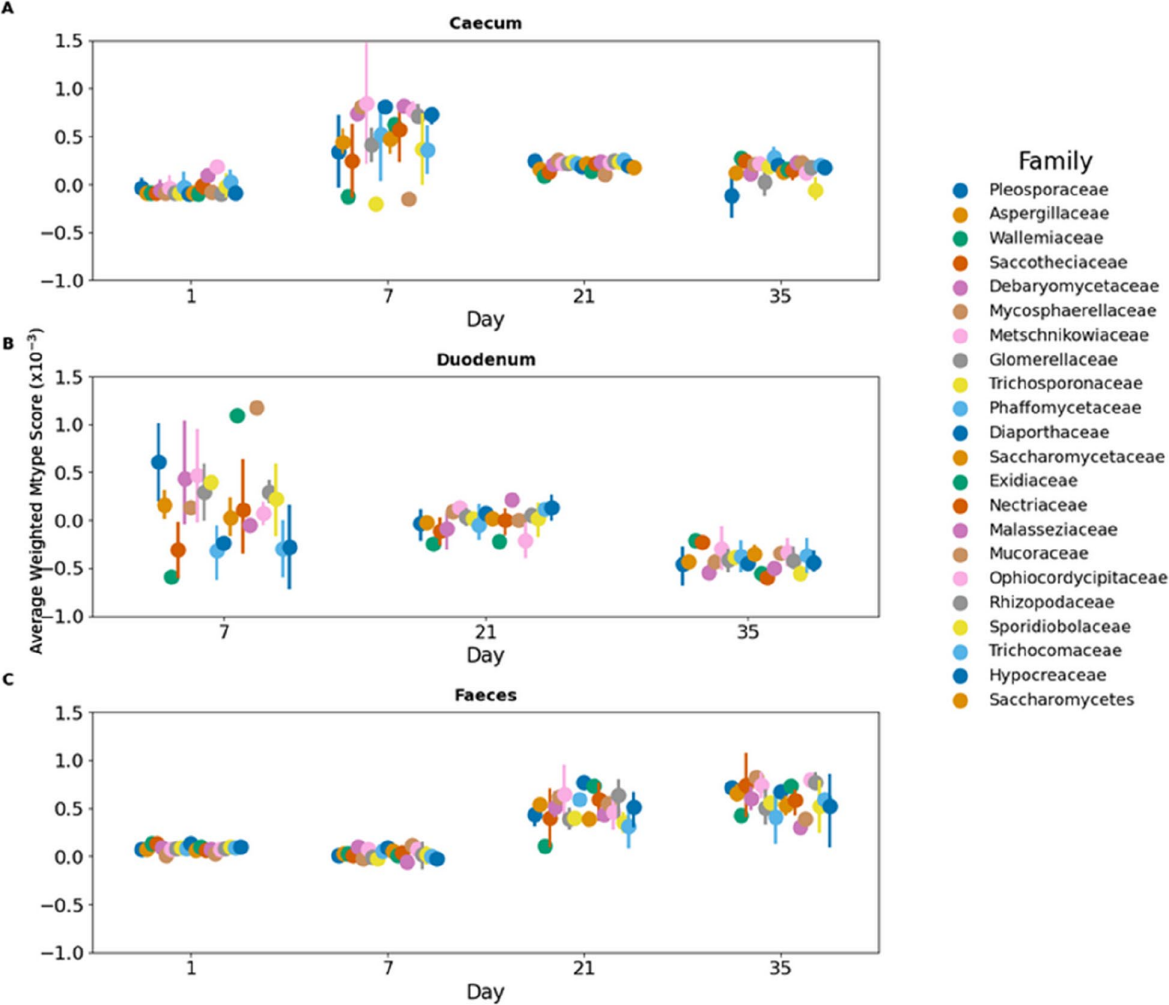
Temporal and gut section distribution of fungal impact on microbial communities. Each point represents an average impact of a fungal family on the microbial community over 35 days. Each panel is a different gut section. Error bars (standard error of the mean) indicate variability within each family across the sampled population

As described previously, the introduced fungi were observed to be able to influence the microbiota (Eq. 2). A general trend of positive effect on faecal microbiota developed, with efficacy depending on the sample (Fig. 4).

**Fig. 5:**
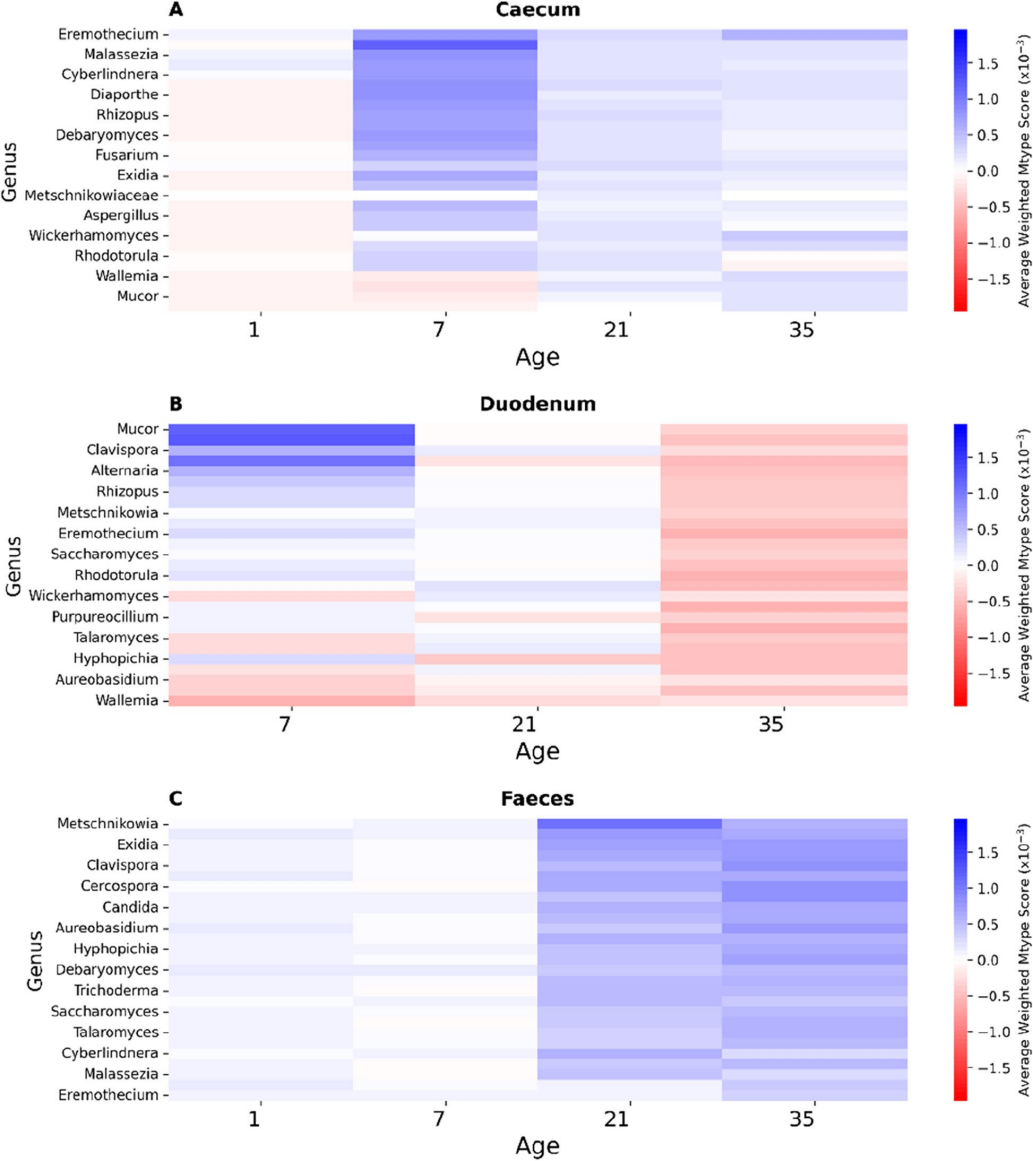
Heatmap depicting the impact of the most positive fungal genera on microbial communities across different gut sections (**A** Caecum, **B** Duodenum, **C** Faeces) and time points (days 1, 7, 21, and 35) in broiler chickens. The colour scale represents the average weighted Mtype score, with positive values (blue) indicating beneficial, negative values (red) indicating a detrimental effect, and white representing a neutral effect. Fungal genera are sorted based on their overall average Mtype score, with the most positively influencing genera at the top of each panel

The genus-level heatmap (Fig. 5) reveals distinct patterns of fungal impact on microbial communities across different gut sections and time points. In the caecum (Fig. 5A), fungal genera such as *Eremothecium*, *Malassezia*, and *Cyberlindera* consistently demonstrate a positive influence on the microbiota (blue), while genera such as *Mucor* show a determinantal effect. However, this is reversed in the Duodenum (Fig. 5B) on day 7, where *Mucor* is one of the most positive genera.

The faecal section (Fig. 5C) displays a shift in fungal impact over time, where fungal genera have a more positive influence with time, with *Metschnikowia* and *Exidia* having the most positive impact.

**Fig. 6:**
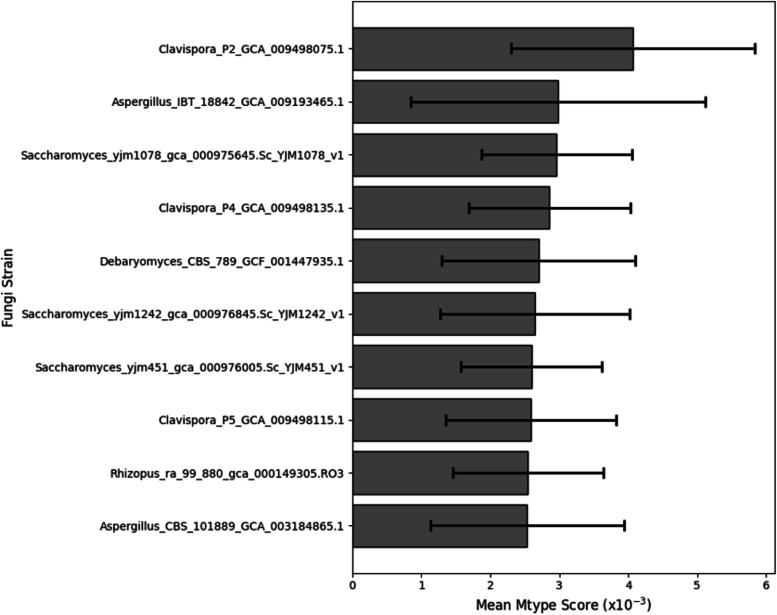
Averaged weighted Mtype score for the top 10 fungi, rated on their potential probiotic impact on poultry microbiota. Higher positive values suggest a more beneficial effect in promoting probiotic bacteria and suppressing pathogenic strains. Error bars represent standard error of the mean

The top 10 fungal strains with the most positive influence on the poultry microbiome included recurring strains from genera such as *Clavispora*, *Aspergillus*, and *Saccharomyces* (Fig. 6).

**Fig. 7:**
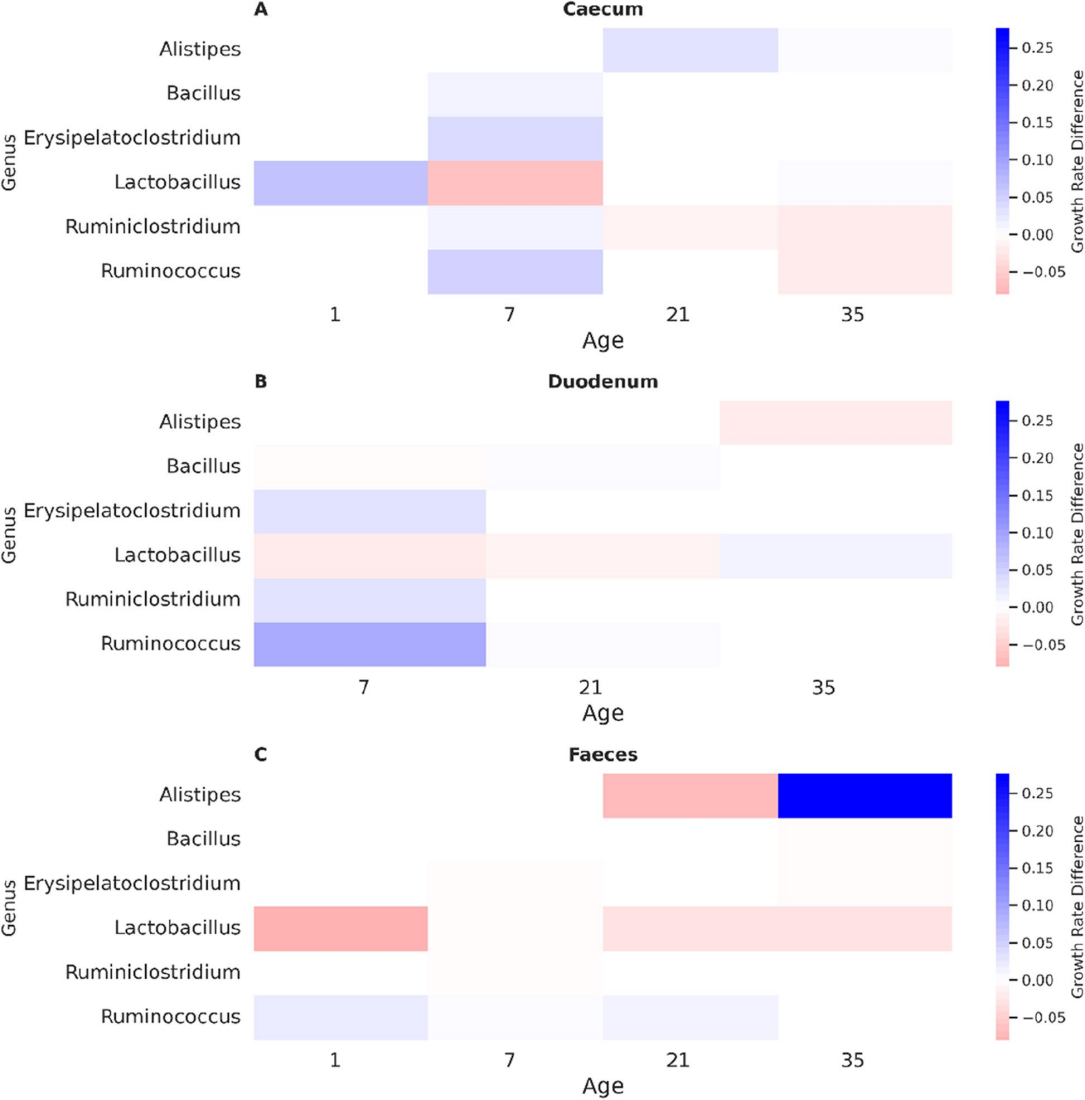
The impact of Clavispora lusitaniae (P2 GCA 009498075) on the growth rate of bacterial genera of each microbiome across gut sections and age. Blue indicates growth promotion; red indicates inhibition due to the addition of C. lusitaniae. Intensity corresponds to the effect of magnitude. Differential effects can be seen for the same genera across different microbiomes

*Clavispora lusitaniae* (P2 GCA 00948075), one of the top-performing strains, demonstrated context-dependent interactions with bacterial genera (Fig. 7). Inhibiting *Alistipes* in the faecal microbiome at age 21 but promoting *Alistipes* at age 35, under the same diet relative to the control.

The impact of diet on the ability of fungi to modulate the microbiome was investigated by comparing mean Mtype scores of introduced fungi under two similar diets (Table 1). The specific strain and dietary context led to notable variations in the impact of these strains on the microbial community.

**Table 1:** Composition of diets used in simulations. Diet 1 represents a standard corn/soy finisher diet [137]. Diet 2 was derived from Liao et al. [53]

Ingredients	Diet 1 (100 g^−1^)	Diet 2 (100 g^−1^)
Corn	70	56.7
Soybean meal	26	34.8

**Fig. 8:**
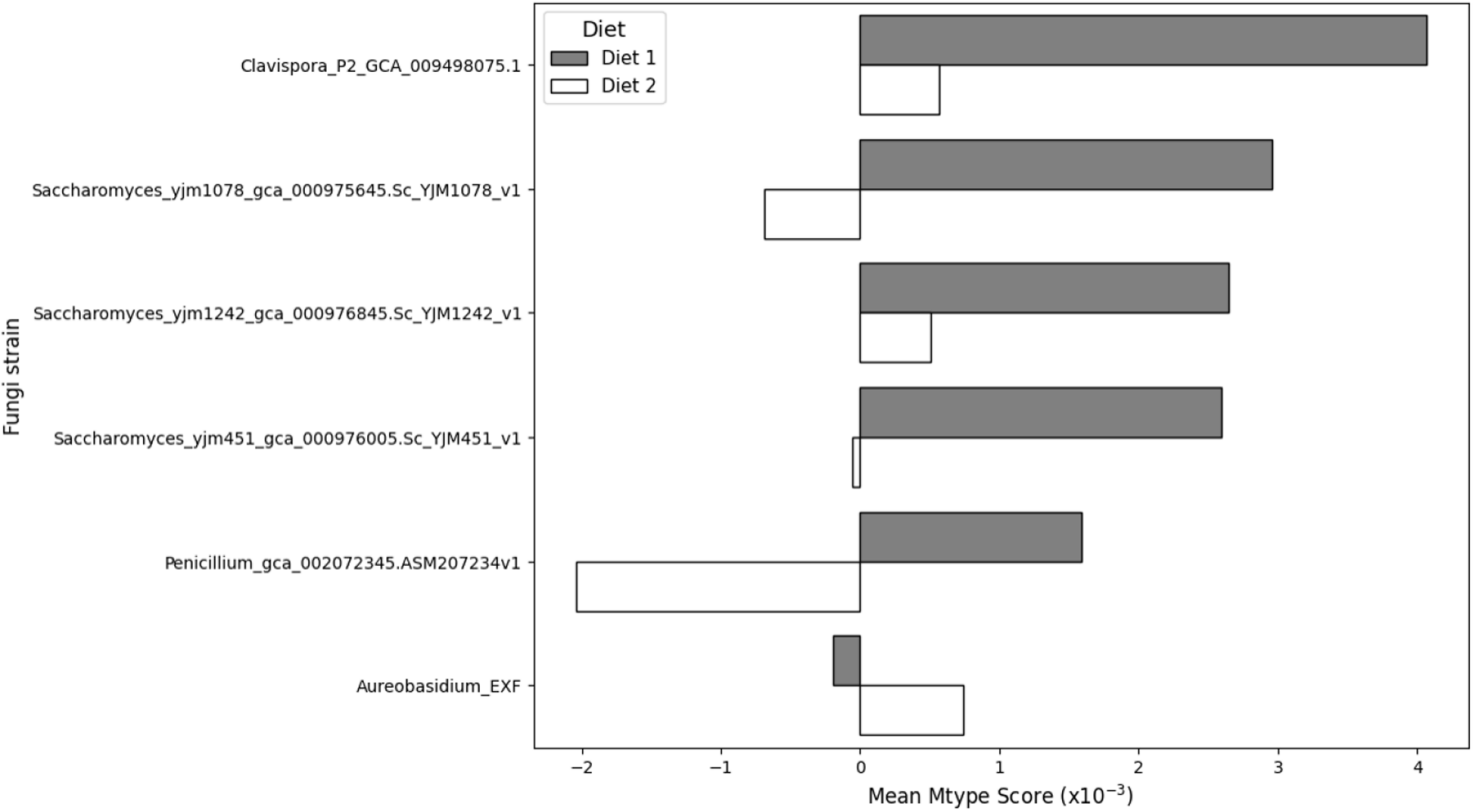
Comparison of the mean Mtype scores for various fungal strains under two diets. This bar chart illustrates the influence of Diet 1 (grey) and Diet 2 (white) on the Mtype scores

Clavispora_P2_GCA_009498075.1, a top-performing strain, exhibited a lower Mtype score under Diet 2 compared to Diet 1, falling below the score of Aureobasidium_EXF, a strain with a previously demonstrated negative Mtype effect. While showing variation within the range of most positive and most negative performing strains, the three *Saccharomyces cerevisiae* strains (Saccharoymces_yjm1078, Saccharomyces_yjm1242, and Saccharomyces_yjm451) also displayed inconsistent responses to even marginal dietary changes, with each strain showing markedly divergent responses in mean Mtype score when exposed to Diet 1 or Diet 2 (Fig. 8).

#### Microbial diversity

The Shannon diversity index was used to quantify the diversity of organisms within the simulated microbial communities. Microbiomes with high diversity are considered more robust and resilient, offering protection against colonisation by opportunistic microbes.

Introducing a probiotic into a highly connected microbial network can cause fluctuations in diversity. A dominating inhibitory effect on select microbes may decrease diversity, while a probiotic that evenly increases the growth of many microbes may enhance diversity.

Artificially introduced fungi showed reduced growth rates in more diverse microbiomes, suggesting that introducing a probiotic within an already diverse environment is challenging due to niche occupancy and increased competition (Fig. 8). The scatterplot illustrates the growth rates of various fungi when introduced to poultry gut microbiota of differing diversity levels, as quantified by the Shannon index (Eq. 3). The linear regression line (*y* = 17 − 0.03x) indicates a moderate inverse relationship (*R* = − 0.6) that is highly significant (*p* < 0.05).

**Fig. 9:**
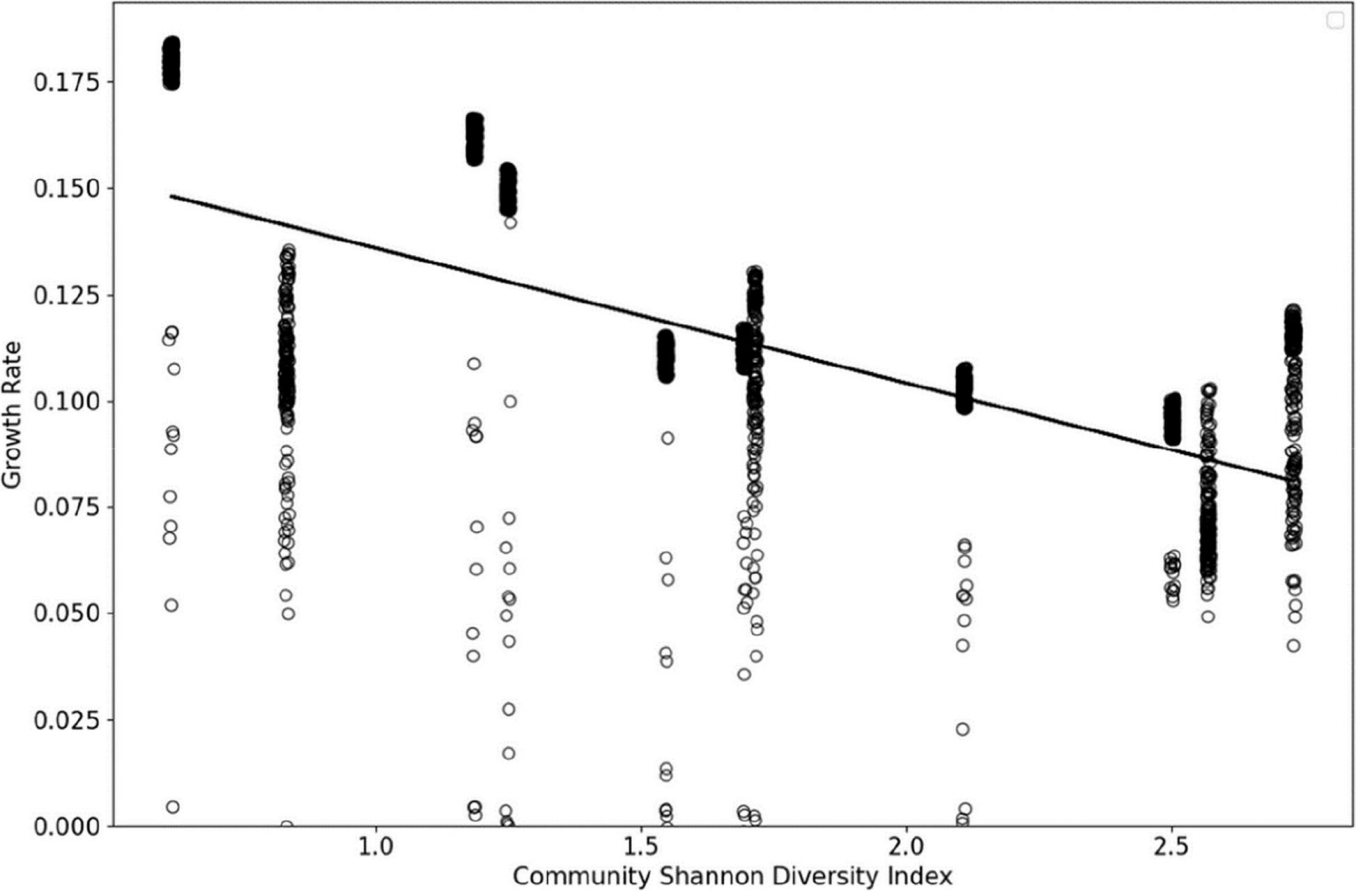
Negative correlation between fungal growth rates and community diversity in poultry gut microbiota. The scatterplot illustrates the growth rates of various fungi when introduced to poultry gut microbiota of differing diversity levels, as quantified by the Shannon index. The linear regression line (*y* = 0.17 − 0.03x) indicates a moderate inverse relationship (*R* = − 0.6) that is highly significant (*P* = 3.21e − 179), suggesting that higher microbial diversity may inhibit fungal growth

**Fig. 10:**
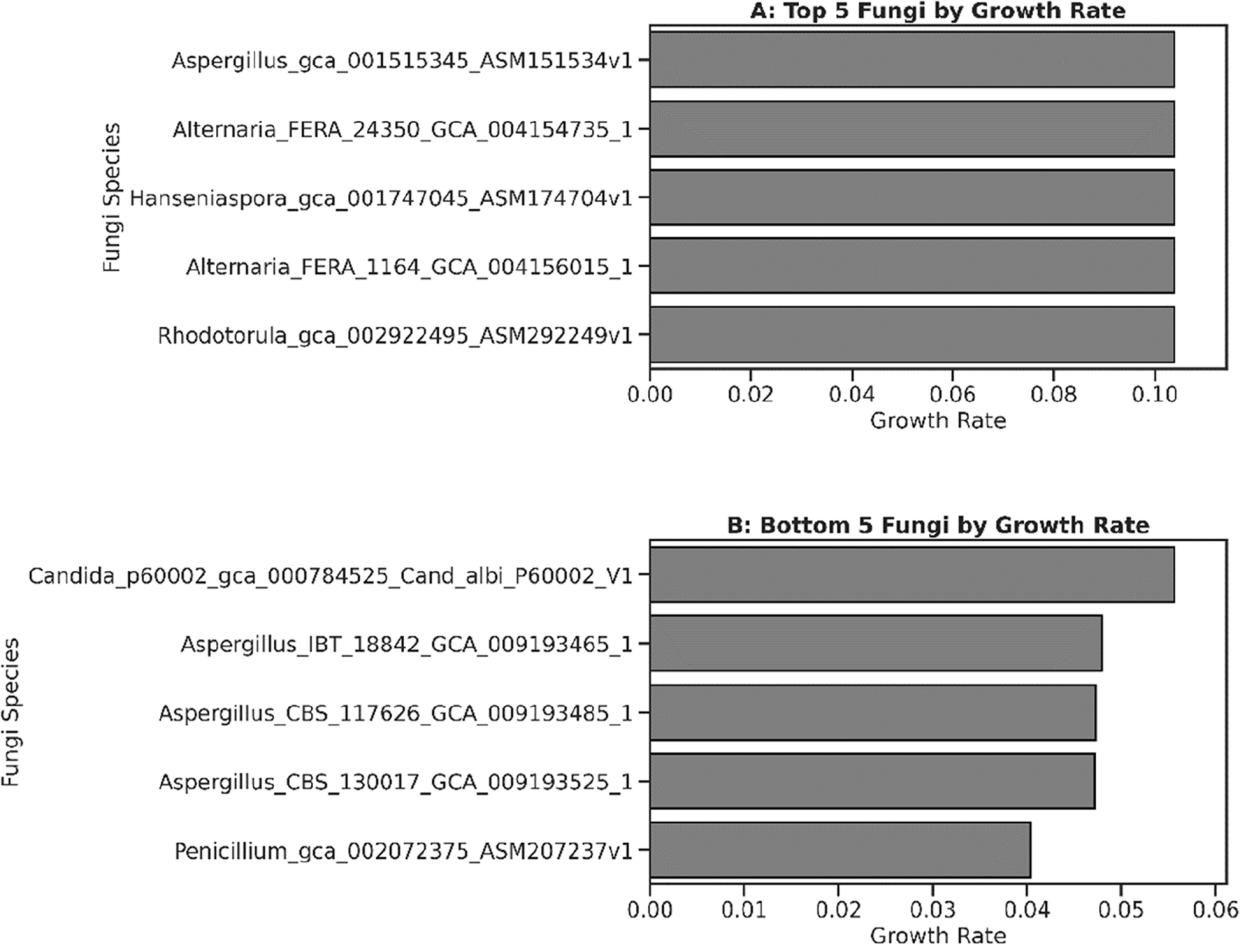
Comparative growth rates of fungi in high-diversity microbiota environments (Shannon index > 2.7). **A** depicts the top five fungal species with the highest growth rates, indicating a potential to thrive in already established complex microbial ecosystems. **B** conversely shows species with the lowest growth rates, showing the range of potential between the fungi examined

Fungi capable of sustaining high growth rates within highly diverse environments (Shannon index > 2.7) could be interesting for probiotic development. The top-performing fungi exhibited notable uniformity in their growth rates (Fig. 9), which could be due to an artefact of the carving process in CarveFungi, which may yield conservative estimations by constraining the metabolic capabilities of the modelling organisms due to limitations in the pan-genomic metabolic model. Furthermore, the bottom five Fungi had less uniform growth rates; however, in the most diverse environments, none of the fungi had a growth rate of 0, as seen in Fig. 10.

Post-normalisation, the Shannon index was calculated using the previously described method (Eq. 3). Fungi that elevate the Shannon index may be considered prime candidates for probiotics, as they promote a more stable microbial community. Conversely, fungi that diminish diversity may dominate the microbiome.

**Fig. 11:**
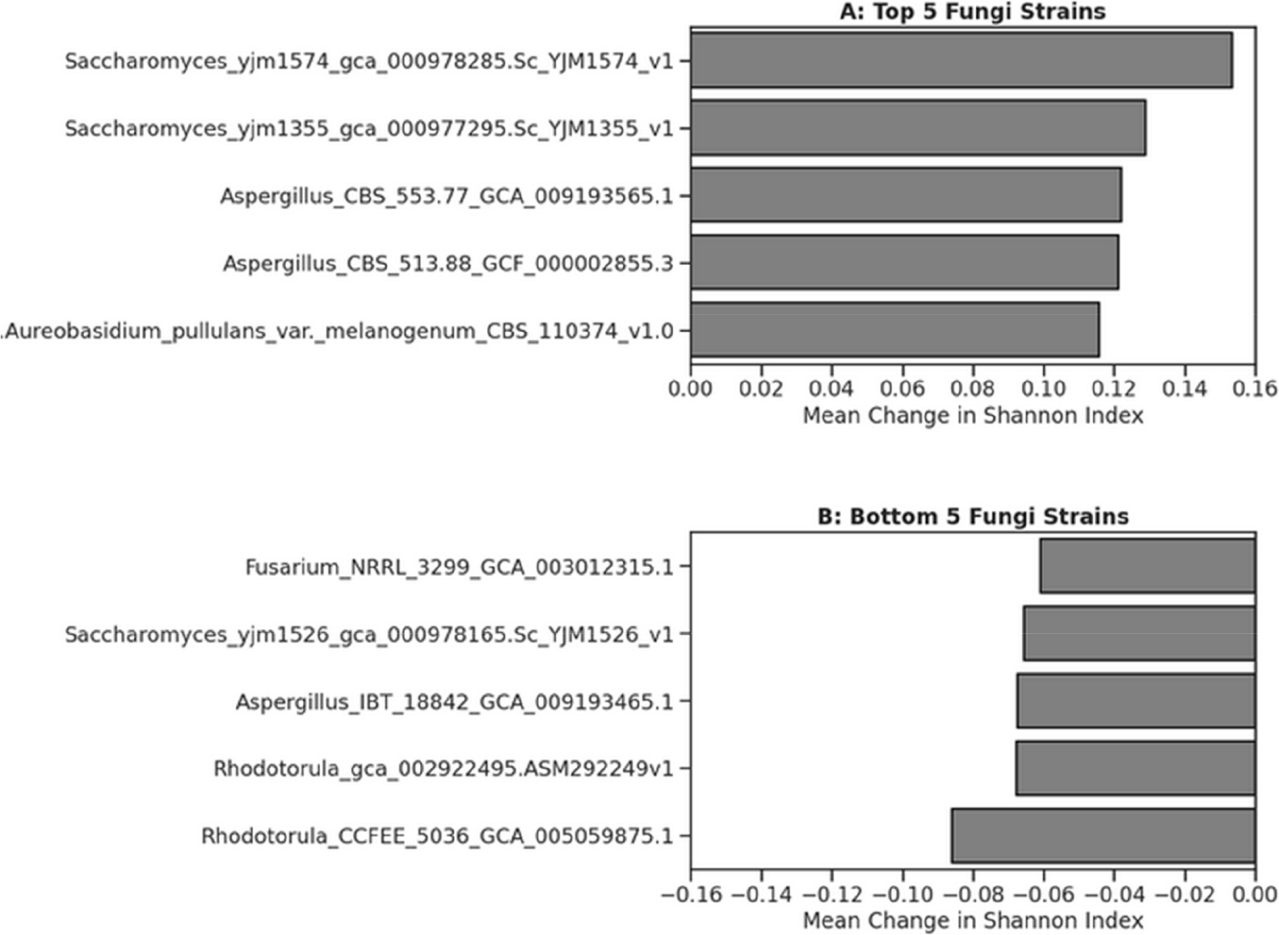
Differential impact of fungal strains on microbial community diversity as a function of growth rates. **A** displays the top five fungal strains with the largest mean positive effect on the Shannon Diversity index, potentially indicating their role in promoting balanced microbial growth. **B** lists the bottom five fungi, showing that some fungi can potentially reduce the Shannon index by competitively excluding/promoting certain microbial species

Different *Saccharomyces cerevisiae* strains exhibited contrasting effects on Diversity (Fig. 10). Stains YJM1574 and YJM1355 (Fig. 11A), isolated from wine and molasses, respectively, occupied the top spots among strains that increased the Shannon index. Conversely, strain YJM1526 (Fig. 11B), a clinical isolate from a throat sample, was among the strains with the most significant decrease in the Shannon index.

### Disease models

**Fig. 12:**
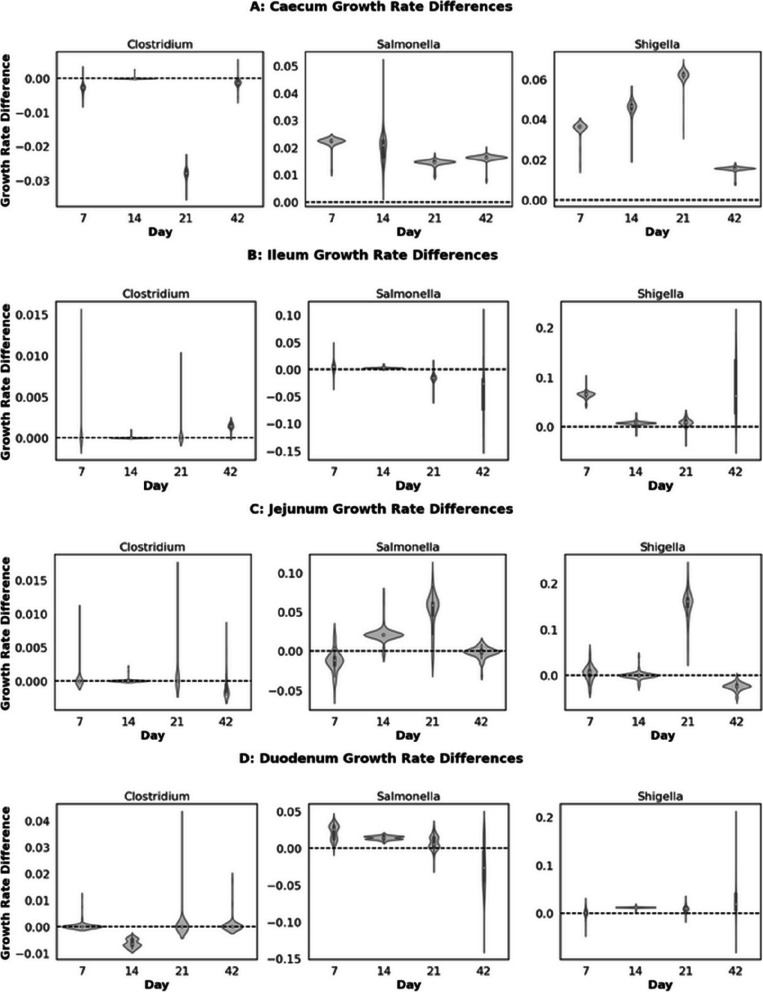
Impact of all fungi presence on growth rate of Clostridium, Salmonella, Shigella in different metagenomic environment Caecum (**A**), Ileum (**B**), Jejunum (**C**), and Duodenum (**D**) of birds that are different ages (7, 14,21, and 42), data points represent the mean growth rate difference between control and probiotic treatments of pathogens within each gut section or age

The growth of commercially relevant poultry pathogens, including Salmonella, Clostridium, and Shigella, was assessed in the presence of potentially probiotic fungi. Pathogens were added at an inclusion of 0.1 to metagenomic samples from the ileum, duodenum, and caecum, alongside potentially probiotic fungi at an inclusion of 0.05. The growth rate of each pathogen was compared to a control without any fungi, and the mean growth rate of each pathogen in each gut section and day was calculated (Fig. 12).

**Fig. 13:**
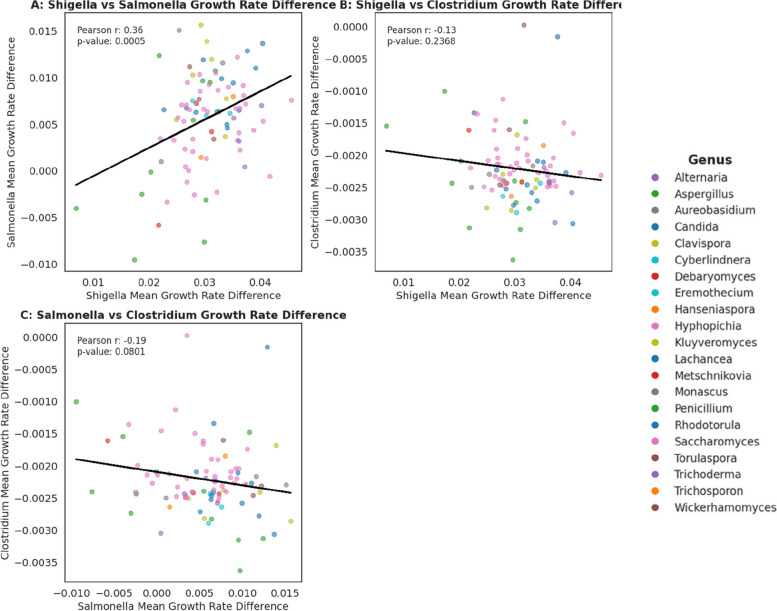
Correlation of mean growth rate differences between Shigella and Salmonella (**A**), Shigella and Clostridium (**B**), Salmonella and Clostridium (**C**). The colour-coded points represent yeast strains that belong to the same genera. Pearson correlation and respective *p* value indicate the extent of significance and correlation between mean growth rate differences

A statistically significant correlation was observed between the gram-negative, rod-shaped Shigella and Salmonella in the presence of various fungal species (Pearson *r* = 0.35, *p* = 0.0015), indicating a broadly consistent response to fungal interaction. In contrast, no significant correlations were found when comparing *Shigella* and *Clostridium* (Pearson *r* = − 0.08, *p* = 0.5032) or *Salmonella* and *Clostridium* (Pearson *r* = − 0.016, *p* = 0.1526) (Fig. 13). Specific fungal interactions of different strains, particularly *Saccharomyces*, did not appear to cluster and were widely spread, suggesting that pathogen-fungi interactions are likely to be strain-specific in the context of these models. However, if a strain could inhibit *Shigella*, it could also inhibit *Salmonella*.

**Fig. 14:**
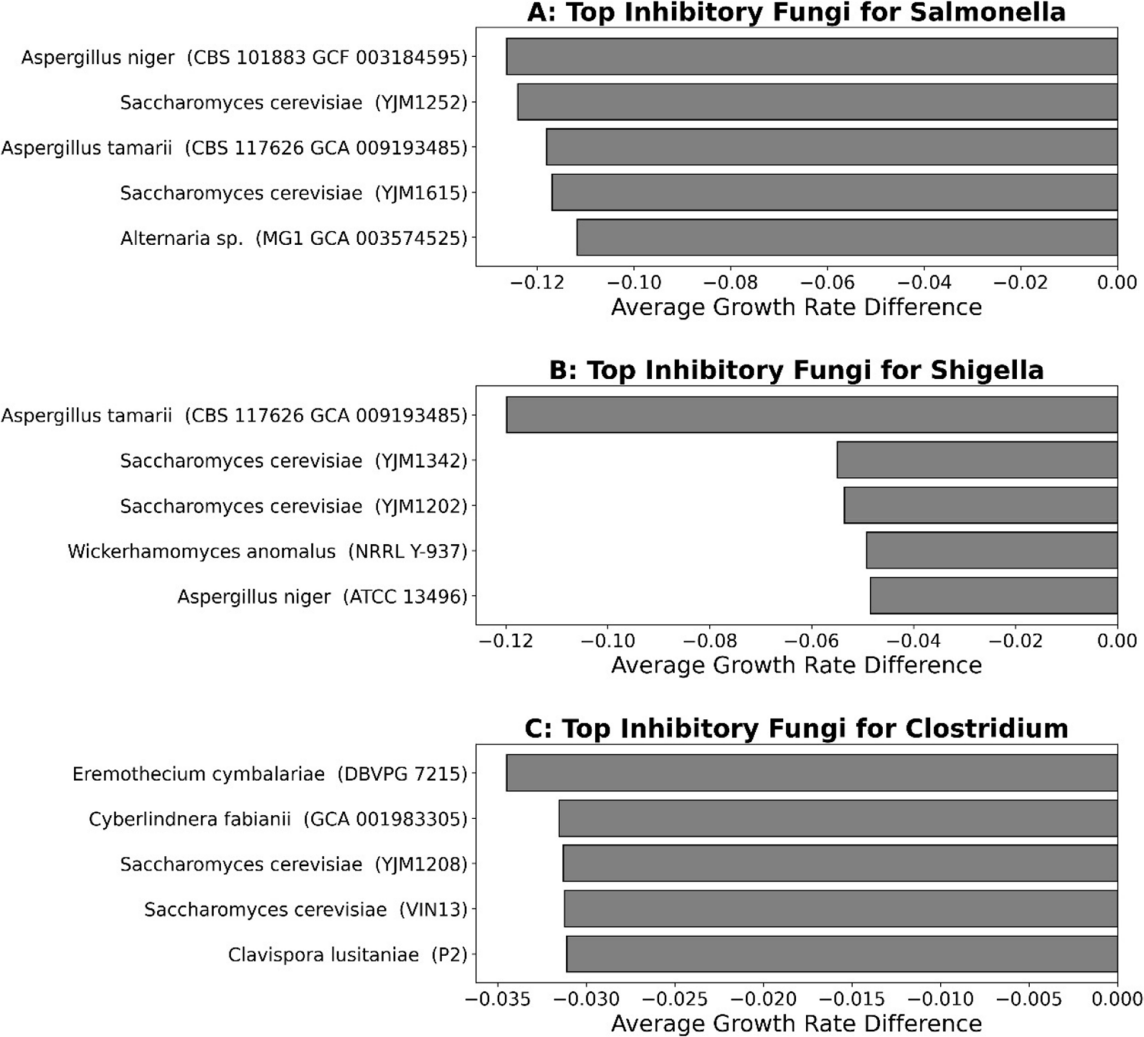
Comparative analysis of fungi inhibition on pathogen growth rates. The addition of fungi shows the most inhibitory average growth rate difference between **A** Salmonella, **B** Shigella, and **C** Clostridium

The top 5 fungi were identified, which resulted in the most considerable mean growth rate difference for each pathogen (Fig. 14).

## Discussion

One of the key findings of our study is the highly context-dependent impact of fungi on the gut microbiome, varying substantially with factors such as the existing microbiome composition and diet (Figs. 7 and 8). This context-dependency presents a significant challenge in identifying universally effective probiotics. The effects of probiotics can differ markedly across different environments. Our results align with previous findings on the individualised responses to probiotics [196] and the influence of diet on probiotic efficacy [197, 198]. This underscores the need for personalised approaches in using metabolic models for probiotic applications tailored to each poultry information, considering the unique characteristics of each host’s microbiome and dietary context [46]. Depending on the commercial poultry operations, if the litter is carried over from one flock to the next, this can serve as an inoculum for the incoming flock [199, 200]. Contrastingly, fresh litter chicks have developed a significantly different microbiome than chicks where litter is reused [201–204]. Therefore, the microbiome's stability would need to be established within each environment.

We validated our metabolic modelling approach by predicting short-chain fatty acid prediction (SCFA) production in the poultry gut (Table 2, Fig. 3). The accuracy of SCFA predictions varied across metabolites and gut sections, with the most robust agreement in the caecum, the primary site of microbial fermentation [205]. Notably, the closest agreement was found when using a more representative diet and a trade-off value of 0.7, highlighting the importance of setting the appropriate parameters to resemble in vivo conditions closely. This could be further improved by having metabolomic data of each gut section at different time points to determine nutrient availability. These findings are consistent with previous studies demonstrating the influence of dietary inputs on SCFA production [206, 207] and the successful prediction of propionate and butyrate in vitro and ex vivo systems [48]. The lower predictive accuracy for acetate may be attributed to the multiple optimal states of overflow metabolism [208, 209], a complex phenomenon that warrants further investigation.

**Table 2:** Pearson coefficient and associated *p* values under different diets and trade-off values, simulations compared to experimental values for Isovalerate, Acetate, Butyrate, Isobutyrate, and Propionate in the Caecum, Duodenum, Ileum, and Jejenum, where Pearson/*p* values (A) and (B) correspond to 0.7 and 0.8, respectively

Simulation	Section	Metabolite	Pearson A	*p* value A	Pearson B	*p* value B
Diet 1	Caecum	Acetate	− 0.348	0.65	0.93	0.02
Diet 1	Jejunum	Acetate	− 0.894	0.3	− 0.89	0.3
Diet 1	Ileum	Acetate	− 0.588	0.41	− 0.58	0.41
Diet 1	Duodenum	Acetate	0.72	0.27	0.72	0.28
Diet 1	Caecum	Butyrate	− 0.06	0.93	− 0.06	0.94
Diet 1	Caecum	Propionate	0.99	0.008	0.99	0.01
Diet 1	Caecum	Isobutyrate	0.85	0.1534	0.84	0.15
Diet 1	Caecum	Isovalerate	0.96	0.04	0.96	0.04
Diet 2	Caecum	Acetate	0.93	0.061	0.35	0.65
Diet 2	Jejunum	Acetate	− 0.95	0.18	− 0.12	0.92
Diet 2	Ileum	Acetate	− 0.09	0.91	− 0.3	0.695
Diet 2	Duodenum	Acetate	0.98	0.01	0.48	0.52
Diet 2	Caecum	Butyrate	0.95	0.04	0.75	0.25
Diet 2	Caecum	Propionate	0.97	0.02	0.99	0.011
Diet 2	Caecum	Isobutyrate	0.97	0.03	0.92	0.078
Diet 2	Caecum	Isovalerate	0.82	0.18	0.95	0.05

Building upon the validation of our metabolic modelling approach, we next examined the strain-specific effects of fungal probiotics on the gut microbiome using Mtype score analysis. Mtype score analysis (Fig. 6) revealed strain-specific and diet-dependant effects of fungal probiotics on the gut microbiome (Fig. 8). The highly variable impact of *Clavispora lusitaniae*, for example, highlights the importance of considering the specific microbiome context when evaluating probiotic candidates (Fig. 5). In one instance, *C. lusitaniae*, suppressed *Alistipes* in the duodenum while promoting it in the faeces of the same age bird (Fig. 7), further illustrating the context-dependent impact of fungi on the microbiome.

Moreover, the observed influence of diet on the direction and magnitude of the probiotic effects illustrates the need for dietary factors in probiotic design and testing. These findings contribute to the growing recognition of the complex interplay between probiotics, the resident microbiome, and host factors such as diet [196–198].

Our analysis of the Shannon diversity index (Figs. 9, 10, and 11) provided insights into the differential effects of fungal strains on microbiome diversity. The introduction of probiotics generally had limited impact in highly diverse, mature microbiomes, consistent with the known challenges of establishing probiotics, and so *in ovo* techniques were established [210–213]. In poultry, microbiome diversity increases from birth, peaks within 14–28 days, and stabilises [214–216], which may contribute to the limited impact of probiotics in mature microbiomes. However, some fungal strains were able to enhance diversity (as determined by our methods), a desirable trait given the association between high diversity and microbiome stability and resilience [217, 218]. Furthermore, introduced probiotic species have been shown to have differential effects on driving diversity changes [219]. These results suggest the development of diversity-promoting probiotics may require careful strain selection and targeting application in less mature or disrupted microbiomes and would be an avenue for further exploration.

Diets 1 and 2 represent very modest changes in composition compared to the differences encountered in commercial practice. For example, in the USA and Latin America, corn and soybean meal are the primary ingredients, while in Europe, wheat is the dominant cereal, and alternative protein sources such as rapeseed meal are more common [220]. If the minor differences between diets 1 and 2 can evoke such a divergent response to a probiotic, the expected response under commercial conditions would be even more divergent. This may explain a great deal of variation noted in the response to probiotics in the literature. The fact that this approach can predict such divergence suggests that metabolic modelling may contribute to probiotics that function consistently across many dietary regimens.

The pathogen inhibition data (Figs. 12 and 13) demonstrated the potential for fungal probiotics to suppress pathogenic bacteria (Fig. 14) selectively and the effect of such to vary depending on strains. However, the inconsistent effects across strains and the specificity of inhibition to specific pathogens further underline the need for targeted probiotic interventions when the need is to target pathogens. The observed correlation does show some generality; however, in responses of Salmonella and Shigella, in contrast to Clostridium, it highlights the importance of considering the specific mechanisms of pathogen inhibition [221], showcasing the need for particular interventions. These findings suggest that probiotic strategies for pathogen control may need to be tailored to the pathogen of interest and validated in the context of the specific microbiome and host environment.

In addition to our study’s focus on using metabolic modelling to identify targeted probiotics, the work of Marinos et al. [222] demonstrates the potential of metabolic modelling to guide the development of precision prebiotics as a complementary approach. Precision prebiotics are compounds that specifically boost the abundance of beneficial microbes already in the microbiome—the work by Marinos et al. [222] shows how metabolic models can produce accurate predictions on effective microbiome modulation.

It is essential to acknowledge its limitations despite the valuable insights it provides into the complex interactions between fungal probiotics, the gut microbiome, and diet. Our approach relies on computational modelling, which, while becoming increasingly powerful, is inherently limited to assumptions and simplifications of the infinitely complex underlying systems. The scope of our study was also necessarily limited to a subset of fungal strains and pathogens, and further work will be needed to assess the practicality of our findings. Moreover, our results must ultimately be validated through in vivo experiments to confirm their biological relevance and applicability.

Despite these limitations, our findings have important implications for developing and applying probiotic interventions in poultry. To our knowledge, this is the first study to apply metabolic modelling for use in poultry and outlines a few potential routes to characterise this approach. Our results highlight the need for more individualised approaches considering specific characteristics of the host microbiome, diet, and disease challenges. The strain-specific effects we observed suggest that probiotic selection may need to move beyond species-level considerations to focus on the unique properties of individual strains. Our work also demonstrates the potential of metabolic modelling to guide the selection of probiotic candidates and to predict the effects on the gut microbiome, opening new avenues for the rational design of targeted interventions.

## Conclusion

In conclusion, our study demonstrates the power of metabolic modelling to elucidate the complex and context-dependent interactions between probiotics and poultry gut-specific microbiomes. We have shown the impact of introduced fungi is highly dependent on the specific strain, resident microbiome composition, and host diet, illustrating the need for targeted, context-specific probiotic approaches. While our work focused on fungal probiotics, the principles and methodologies are broadly applicable. Realising the full potential of this approach will require expanding and refining metabolic species specific to poultry, with genome-scale metabolic models enhanced by integrating transcriptomic data. Furthermore, metabolomic and metagenomic time-series data would not only enable the validation of predicted temporal dynamics and provide insights into the stability and resilience of probiotic interventions but also serve as tools to improve those predictions. Integrating multi-omics data in such a manner provides a systems-level understanding of the complex interactions between probiotics, the gut microbiome, and the host. As the world seeks sustainable alternatives to antibiotic growth promoters, harnessing the power of the gut microbiome through metabolic modelling will be essential. This work represents a significant step towards a new era of precision microbiome management in agriculture, providing a foundation for developing targeted, effective, and sustainable microbiome-based solutions to promote poultry health and productivity. While this work focuses on the poultry industry, the principles and methodologies presented here could also be adapted to other livestock, opening new avenues for farm-specific probiotic interventions.

## Supplementary Information

Supplementary Material 1: Supplementary Table S1: 90 fungal strains selected based on the available literature.

Supplementary Material 2: Supplementary Table S2: A detailed breakdown of digestibility calculations.

Supplementary Material 3: Supplementary Table S3: The net impact of introduced fungi on the microbiome.

Supplementary Material 4.

Supplementary Material 5.

## Data Availability

All Data and scripts are available in the GitHub or in the supplementary files.

## References

[1] Ban on antibiotics as growth promoters in animal feed enters into effect. 2005. Available from: https://ec.europa.eu/commission/presscorner/detail/en/IP_05_1687.

[2] Kirchhelle C. Pharming animals: a global history of antibiotics in food production (1935–2017). Palgrave Commun. 2018;4(1):96.

[3] Singh KS, et al. Antimicrobial resistance dynamics and the one-health strategy: a review. Environ Chem Lett. 2021;19(4):2995–3007.

[4] Van Boeckel TP, et al. Global trends in antimicrobial resistance in animals in low- and middle-income countries. Science. 2019;365(6459):eaaw1944. 31604207 10.1126/science.aaw1944

[5] Directorate VM. Withdrawal of marketing authorisations of veterinary medicines containing Zinc Oxide, V.M. Directorate, Editor. 2022. Available from: https://www.gov.uk/government/organisations/veterinary-medicines-directorate.

[6] Guerreiro F. EU ban on routine farm use of antimicrobials. European parliament. 2021; E-000779/2021.

[7] Gong L, et al. Effects of three probiotic Bacillus on growth performance, digestive enzyme activities, antioxidative capacity, serum immunity, and biochemical parameters in broilers. Anim Sci J. 2018;89(11):1561–71. 30198073 10.1111/asj.13089

[8] Zhen W, et al. Effect of dietary Bacillus coagulans supplementation on growth performance and immune responses of broiler chickens challenged by Salmonella enteritidis. Poult Sci. 2018;97(8):2654–66. 29660095 10.3382/ps/pey119

[9] He T, et al. Effects of probiotics as antibiotics substitutes on growth performance, serum biochemical parameters, intestinal morphology, and barrier function of broilers. Animals. 2019;9(11):985. 31752114 10.3390/ani9110985 PMC6912548

[10] Ebeid TA, Al-Homidan IH, Fathi MM. Physiological and immunological benefits of probiotics and their impacts in poultry productivity. Worlds Poult Sci J. 2021;77(4):883–99.

[11] Liu X, et al. Growth performance and meat quality of broiler chickens supplemented with Bacillus licheniformis in drinking water. Asian-Australas J Anim Sci. 2012;25(5):682–9. 25049614 10.5713/ajas.2011.11334 PMC4093119

[12] Ding S, et al. The impact of probiotics on gut health via alternation of immune status of monogastric animals. Anim Nutr. 2021;7(1):24–30. 33997328 10.1016/j.aninu.2020.11.004 PMC8110871

[13] Metchnikoff E. The prolongation of life : optimistic studies. Putnam, London. 1908.

[14] McFarland LV. From yaks to yogurt: the history, development, and current use of probiotics. Clin Infect Dis. 2015;60(suppl_2):S85–90. 25922406 10.1093/cid/civ054

[15] Khorshidian N, Yousefi M, Mortazavian AM. Chapter three - fermented milk: the most popular probiotic food carrier. In: da Cruz AG, et al., editors. Advances in food and nutrition research. London: Academic Press; 2020. p. 91–114. 10.1016/bs.afnr.2020.06.007 32892839

[16] Ryan KA, et al. Isolation of lactobacilli with probiotic properties from the human stomach. Lett Appl Microbiol. 2008;47(4):269–74. 19241519 10.1111/j.1472-765x.2008.02416.x

[17] Al-Nijir M, Henk DA, Bedford MR, Chuck CJ. Assessing Metschnikowia pulcherrima as a potential probiotic yeast for animal feed. Sustain Micobiol. 2024;1:qvae008. 10.1093/sumbio/qvae008 PMC13409136 42576896

[18] Bermudez-Brito M, et al. Sources, isolation, characterisation and evaluation of probiotics. Br J Nutr. 2013;109(S2):S35–50. 23360880 10.1017/S0007114512004011

[19] De Waard R, et al. Comparison of faecal Lactobacillus populations in experimental animals from different breeding facilities and possible consequences for probiotic studies. Lett Appl Microbiol. 2002;34(2):105–9. 11849504 10.1046/j.1472-765x.2002.01051.x

[20] Zmora N, et al. Personalized gut mucosal colonization resistance to empiric probiotics is associated with unique host and microbiome features. Cell. 2018;174(6):1388-1405.e21. 30193112 10.1016/j.cell.2018.08.041

[21] Pettersen JP, et al. Genome-scale metabolic models reveal determinants of phenotypic differences in non-Saccharomyces yeasts. BMC Bioinformatics. 2023;24(1):438. 37990145 10.1186/s12859-023-05506-7 PMC10664357

[22] Orth JD, Thiele I, Palsson BØ. What is flux balance analysis? Nat Biotechnol. 2010;28(3):245–8. 20212490 10.1038/nbt.1614 PMC3108565

[23] Raman K, Chandra N. Flux balance analysis of biological systems: applications and challenges. Brief Bioinform. 2009;10(4):435–49. 19287049 10.1093/bib/bbp011

[24] Diener C, M Gibbons Sean, O Resendis-Antonio. MICOM: metagenome-scale modeling to infer metabolic interactions in the gut microbiota. mSystems. 2020;5(1). 10.1128/msystems.00606-19. 10.1128/mSystems.00606-19 PMC6977071 31964767

[25] Castillo S, Peddinti G, Blomberg P, Jouhten P. Reconstruction of compartmentalized genome-scale metabolic models using deep learning for over 800 fungi. bioRxiv. 2023:2023.08.23.554328.

[26] Brunner J, Chia N. Metabolic model-based ecological modeling for probiotic design. ELife. 2024;13:e83690. 10.7554/eLife.83690 PMC10942782 38380900

[27] Hamilton R, Proudfoot F. The value of growth promotants in meat birds. Misset-World Poultry. 1991;7:35.

[28] Bradley GL, Savage TF, Timm KI. The effects of supplementing diets with saccharomyces cerevisiae var. boulardii on male poult performance and ileal morphology1. Poult Sci. 1994;73(11):1766–70. 7862617 10.3382/ps.0731766

[29] Miles R, Bootwalla S. Direct-fed microbials in animal production. A review of literature. West Des Moines: Natt. Feed Ingred. Assoc.; 1991.

[30] Stanley VG, et al. The use of Saccharomyces cerevisiae to suppress the effects of aflatoxicosis in broiler chicks. Poult Sci. 1993;72(10):1867–72. 8415359 10.3382/ps.0721867

[31] Ogbuewu IP, et al. Yeast (Saccharomyces cerevisiae) and its effect on production indices of livestock and poultry—a review. Comp Clin Path. 2019;28(3):669–77.

[32] Dantán-González E, et al. Impact of Meyerozyma guilliermondii isolated from chickens against Eimeria sp. protozoan, an in vitro analysis. BMC Vet Res. 2015;11(1):278. 26552648 10.1186/s12917-015-0589-0 PMC4640389

[33] Sugiharto S, et al. Effect of dietary supplementation with Rhizopus oryzae or Chrysonilia crassa on growth performance, blood profile, intestinal microbial population, and carcass traits in broilers exposed to heat stress. Arch Anim Breed. 2017;60(3):347–56.

[34] Sugiharto S, Yudiarti T, Isroli I, Widiastuti E, Wahyuni HI, Sartono TA. The effect of fungi-origin probiotic Chrysonilia crassa in comparison to selected commercially used feed additives on broiler chicken performance, intestinal microbiology, and blood indices. J Adv Vet Anim Res. 2018;5(3):332–42.

[35] Hicks R, et al. The Oleaginous yeast Metschnikowia pulcherrima displays killer activity against avian-derived pathogenic bacteria. Biol. 2021;10:1227. 10.3390/biology10121227 PMC8698481 34943142

[36] Stanke M, Morgenstern B. AUGUSTUS: a web server for gene prediction in eukaryotes that allows user-defined constraints. Nucleic Acids Res. 2005;33(Web Server issue):W465-7. 15980513 10.1093/nar/gki458 PMC1160219

[37] Huerta-Cepas J, et al. eggNOG 5.0: a hierarchical, functionally and phylogenetically annotated orthology resource based on 5090 organisms and 2502 viruses. Nucleic Acids Res. 2019;47(D1):D309-d314. 30418610 10.1093/nar/gky1085 PMC6324079

[38] Buchfink B, Reuter K, Drost H-G. Sensitive protein alignments at tree-of-life scale using DIAMOND. Nat Methods. 2021;18(4):366–8. 33828273 10.1038/s41592-021-01101-x PMC8026399

[39] Buchan DWA, Jones DT. The PSIPRED protein analysis workbench: 20 years on. Nucleic Acids Res. 2019;47(W1):W402-w407. 31251384 10.1093/nar/gkz297 PMC6602445

[40] Bekiaris PS, Klamt S. Automatic construction of metabolic models with enzyme constraints. BMC Bioinformatics. 2020;21(1):19. 31937255 10.1186/s12859-019-3329-9 PMC6961255

[41] Chang A, et al. BRENDA, the ELIXIR core data resource in 2021: new developments and updates. Nucleic Acids Res. 2020;49(D1):D498–508. 10.1093/nar/gkaa1025 PMC7779020 33211880

[42] King ZA, et al. BiGG models: a platform for integrating, standardizing and sharing genome-scale models. Nucleic Acids Res. 2015;44(D1):D515–22. 26476456 10.1093/nar/gkv1049 PMC4702785

[43] Wittig U, et al. SABIO-RK: an updated resource for manually curated biochemical reaction kinetics. Nucleic Acids Res. 2017;46(D1):D656–60. 10.1093/nar/gkx1065 PMC5753344 29092055

[44] Consortium TU. UniProt: the universal protein knowledgebase in 2023. Nucleic Acids Res. 2022;51(D1):D523–31. 10.1093/nar/gkac1052 PMC9825514 36408920

[45] Scott WT Jr, et al. A structured evaluation of genome-scale constraint-based modeling tools for microbial consortia. PLoS Comput Biol. 2023;19(8):e1011363. 37578975 10.1371/journal.pcbi.1011363 PMC10449394

[46] Carr A, Baliga NS, Diener C, Gibbons SM. Personalized Clostridioides difficile engraftment risk prediction and probiotic therapy assessment in the human gut. bioRxiv. 2023:2023.04.28.538771. 10.1016/j.cels.2025.101367 PMC12497417 40774255

[47] Gibbons SM, et al. Perspective: leveraging the gut microbiota to predict personalized responses to dietary, prebiotic, and probiotic interventions. Adv Nutr. 2022;13(5):1450–61. 35776947 10.1093/advances/nmac075 PMC9526856

[48] Quinn-Bohmann N, Wilmanski T, Sarmiento KR, Levy L, Lampe JW, Gurry T, Rappaport N, Ostrem EM, Venturelli OS, Diener C, Gibbons SM.&nbsp;Microbial community-scale metabolic modeling predicts personalized short chain fatty acid production profiles in the human gut. bioRxiv. 2023. 10.1038/s41564-024-01728-4 PMC11841136 38914826

[49] Heinken A, et al. Genome-scale metabolic reconstruction of 7,302 human microorganisms for personalized medicine. Nat Biotechnol. 2023;41(9):1320–31. 36658342 10.1038/s41587-022-01628-0 PMC10497413

[50] Fanelli MJ, et al. Localization of salmonellae within the intestinal tract of chickens. Avian Dis. 1971;15(2):366–75. 4932189

[51] Stamilla A, et al. Analysis of the microbial intestinal tract in broiler chickens during the rearing period. Biol. 2021;10(9):942. 10.3390/biology10090942 PMC8469170 34571819

[52] Craven SE. Colonization of the intestinal tract by clostridium perfringens and fecal shedding in diet-stressed and unstressed broiler chickens. Poult Sci. 2000;79(6):843–9. 10875766 10.1093/ps/79.6.843

[53] Liao X, et al. The relationship among gut microbiota, short-chain fatty acids, and intestinal morphology of growing and healthy broilers. Poult Sci. 2020;99(11):5883–95. 33142506 10.1016/j.psj.2020.08.033 PMC7647869

[54] AlMatar M, Var I, Koksal F. How does alternaria alternata-derived alternariol affect our health? Mini Rev Org Chem. 2016;13(6):465–72.

[55] Puvača N, et al. Optical characterization of Alternaria spp. contaminated wheat grain and its influence in early broilers nutrition on oxidative stress. Sustainability. 2021;13(7):4005.

[56] Arastehfar A, et al. Multidrug-resistant Trichosporon species: underestimated fungal pathogens posing imminent threats in clinical settings. Crit Rev Microbiol. 2021;47:679. 34115962 10.1080/1040841X.2021.1921695

[57] Hu X, et al. A new mycoparasite, Aspergillus sp. ASP-4, parasitizes the sclerotia of Sclerotinia sclerotiorum. Crop Prot. 2013;54:15–22.

[58] Varga J, Frisvad JC, Samson RA. Two new aflatoxin producing species, and an overview of Aspergillus section Flavi. Stud Mycol. 2011;69(1):57–80. 21892243 10.3114/sim.2011.69.05 PMC3161756

[59] Saleh A, et al. Effects of feeding aspergillus awamori and aspergillus niger on growth performance and meat quality in broiler chickens. J Poult Sci. 2011;48:201–6.

[60] Peterson SW, et al. Aspergillus bombycis, a new aflatoxigenic species and genetic variation in its sibling species, A. nomius. Mycologia. 2001;93(4):689–703.

[61] Alshehri B, Palanisamy M. Evaluation of molecular identification of Aspergillus species causing fungal keratitis. Saudi J Biol Sci. 2020;27(2):751–6. 32210696 10.1016/j.sjbs.2019.12.030 PMC6997875

[62] Le Pape P, et al. First case of Aspergillus caelatus airway colonization in a Chronic Obstructive Pulmonary Disease patient. Int J Infect Dis. 2019;81:85–90. 30690215 10.1016/j.ijid.2019.01.043

[63] Queiroz B, et al. Fungal contamination and determination of fumonisins and aflatoxins in commercial feeds intended for ornamental birds in Rio de Janeiro. Brazil Lett Appl Microbiol. 2013;57(5):405–11. 23815153 10.1111/lam.12127

[64] Elaroussi MA, et al. Experimental ochratoxicosis in broiler chickens. Avian Pathol. 2006;35(4):263–9. 16854637 10.1080/03079450600817115

[65] Fernandez-Pittol M, et al. Aspergillosis by cryptic Aspergillus species: a case series and review of the literature. Rev Iberoam Micol. 2022;39(2):44–9. 35753971 10.1016/j.riam.2022.04.002

[66] Massi FP, et al. Prospecting for the incidence of genes involved in ochratoxin and fumonisin biosynthesis in Brazilian strains of Aspergillus niger and Aspergillus welwitschiae. Int J Food Microbiol. 2016;221:19–28. 26803270 10.1016/j.ijfoodmicro.2016.01.010

[67] Arné P, et al. Aspergillus fumigatus in Poultry. Int J Microbiol. 2011;2011:746356. 21826144 10.1155/2011/746356 PMC3150149

[68] Filipe D, et al. Solid-State fermentation of distiller’s dried grains with solubles improves digestibility for European Seabass (Dicentrarchus labrax) Juveniles. Fishes. 2023;8(2):90.

[69] Alagawany M, et al. Use of Aspergillus japonicas culture filtrate as a feed additive in quail breeder’s nutrition. Ital J Anim Sci. 2020;19(1):1289–96.

[70] Zhang X, Jiao R, Li H, Ou D, Zhang D, Shen Y, Ling N, Ye Y. Probiotic Potential, Antibacterial, and Antioxidant Capacity of Aspergillus luchuensis YZ-1 Isolated From Liubao Tea. Probiotics Antimicrob Proteins. 2024;5:1528–40. 10.1007/s12602-023-10126-x 37458925

[71] KyungWoo L, Lee S, Lee B. Aspergillus oryzae as probiotic in poultry - a review*.* Int J Poult Sci. 2006;5:1–3.

[72] Fouad AM, et al. Harmful effects and control strategies of aflatoxin B1 produced by Aspergillus flavus and Aspergillus parasiticus strains on poultry: review. Toxins (Basel). 2019;11(3):176. 30909549 10.3390/toxins11030176 PMC6468546

[73] El-Sharkawy H, Abd El-Salam AM, Tahoun A. Pathology and epidemiology of fungal infections in layer chicken flocks. Adv Gut Microbiome Res. 2023;2023:9956074.

[74] Ito Y, et al. Aspergillus pseudotamarii, a new aflatoxin producing species in Aspergillus section Flavi. Mycol Res. 2001;105(2):233–9.

[75] Frisvad JC, Skouboe P, Samson RA. Taxonomic comparison of three different groups of aflatoxin producers and a new efficient producer of aflatoxin B1, sterigmatocystin and 3-O-methylsterigmatocystin, Aspergillus rambellii sp. nov. Syst Appl Microbiol. 2005;28(5):442–53. 16094871 10.1016/j.syapm.2005.02.012

[76] Ostry V, Malir F, Ruprich J. Producers and important dietary sources of ochratoxin a and citrinin. Toxins (Basel). 2013;5(9):1574–86. 24048364 10.3390/toxins5091574 PMC3798874

[77] Frisvad JC, et al. Taxonomy of Aspergillus section Flavi and their production of aflatoxins, ochratoxins and other mycotoxins. Stud Mycol. 2019;93(1):1–63. 30108412 10.1016/j.simyco.2018.06.001 PMC6080641

[78] Balajee SA. Aspergillus terreus complex. Med Mycol. 2009;47(sup1):S42–6. 19291598 10.1080/13693780802562092

[79] Rizwan M, et al. Aspergillosis: an occupational zoonotic disease. Zoonosis, Unique Scientific Publishers, Faisalabad, Pakistan. 2023;4:380–91.

[80] Gautier M, et al. Aspergillus tubingensis : a major filamentous fungus found in the airways of patients with lung disease. Med Mycol. 2016;54(5):459–70. 26773134 10.1093/mmy/myv118

[81] Gyotoku H, et al. A case of bronchial aspergillosis caused by Aspergillus udagawae and its mycological features. Med Mycol. 2012;50(6):631–6. 22149972 10.3109/13693786.2011.639036

[82] Perrone G, et al. Aspergillus uvarum sp. nov., an uniseriate black Aspergillus species isolated from grapes in Europe. Int J Syst Evol Microbiol. 2008;58(4):1032–9. 18398215 10.1099/ijs.0.65463-0

[83] Chinajariyawong C, Muangkeow N. Carcass yield and visceral organs of broiler chickens fed palm kernel meal or Aspergillus wentii TISTR 3075 fermented palm kernel meal. Walailak Journal of Science and Technology (WJST). 2011;8(2):175–85.

[84] Zajc J, et al. From glaciers to refrigerators: the population genomics and biocontrol potential of the black yeast Aureobasidium subglaciale. Microbiology Spectrum. 2022;10(4):e01455-e1522. 35880866 10.1128/spectrum.01455-22 PMC9430960

[85] Rhimi W, et al. Virulence and in vitro antifungal susceptibility of Candida albicans and Candida catenulata from laying hens. Int Microbiol. 2021;24(1):57–63. 32772220 10.1007/s10123-020-00141-1 PMC7873078

[86] Lim SJ, et al. Opportunistic yeast pathogen Candida spp.: Secreted and membrane-bound virulence factors. Med Mycol. 2021;59(12):1127–44. 34506621 10.1093/mmy/myab053

[87] Wong HM, et al. The first case report of septic arthritis and osteomyelitis of the knee caused by Candida viswanathii. Infect Dis Clin Pract. 2020;28(2):109–11.

[88] Simões LA, et al. Probiotic properties of yeasts isolated from Brazilian fermented table olives. J Appl Microbiol. 2021;131(4):1983–97. 33704882 10.1111/jam.15065

[89] Rangel LI, et al. Cercospora beticola: the intoxicating lifestyle of the leaf spot pathogen of sugar beet. Mol Plant Pathol. 2020;21(8):1020–41. 32681599 10.1111/mpp.12962 PMC7368123

[90] Hernandez AP, et al. An in-field heat treatment to reduce Cercospora beticola survival in plant residue and improve Cercospora leaf spot management in sugarbeet. Front Plant Sci. 2023;14:1100595. 37229110 10.3389/fpls.2023.1100595 PMC10204640

[91] Satora P, et al. Yeast microbiota during sauerkraut fermentation and its characteristics. Int J Mol Sci. 2020;21(24):9699. 33353237 10.3390/ijms21249699 PMC7767181

[92] Paniz-Mondolfi AE, et al. First report of human infection caused by Colletotrichum chlorophyti occurring in a post-corneal transplant patient with endophthalmitis. Medical Mycology Case Reports. 2021;32:73–6. 33996426 10.1016/j.mmcr.2021.04.002 PMC8102205

[93] Gao M, et al. Molecular and physiological characterization of Arabidopsis-Colletotrichum gloeosporioides pathosystem. Plant Pathol. 2021;70(5):1168–79.

[94] Oliveira Silva AD, et al. Fungal pathogenesis-related cell wall biogenesis, with emphasis on the maize anthracnose fungus Colletotrichum graminicola. Plants. 2022;11(7):849. 35406829 10.3390/plants11070849 PMC9003368

[95] Yan Y, et al. Colletotrichum higginsianum as a model for understanding host-pathogen interactions: a review. Int J Mol Sci. 2018;19(7):2142. 30041456 10.3390/ijms19072142 PMC6073530

[96] Yang H-C, Haudenshield JS, Hartman GL. Colletotrichum incanum sp. nov., a curved-conidial species causing soybean anthracnose in USA. Mycologia. 2014;106(1):32–42. 24603833 10.3852/13-013

[97] Kubo Y, et al. Development of the infection strategy of the hemibiotrophic plant pathogen, Colletotrichum orbiculare, and plant immunity. Physiol Mol Plant Pathol. 2016;95:32–6.

[98] Charron C, et al. Characterization of Colletotrichum orchidophilum, the agent of black spot disease of vanilla. J Phytopathol. 2018;166(7–8):525–31.

[99] Amaral Carneiro G, Storti A, Baric S. First report of colletotrichum salicis causing bitter rot of apple in Italy. Plant Dis. 2021;105(1):224.

[100] Fujinaga M, et al. First report of celery stunt anthracnose caused by Colletotrichum simmondsii in Japan. J Gen Plant Pathol. 2011;77(4):243–7.

[101] Xu J, et al. Pathogenic variability of isolates of Colletotrichum sublineola on Sorghum in China. Physiol Mol Plant Pathol. 2023;126:102046.

[102] Hiruma K, et al. A fungal sesquiterpene biosynthesis gene cluster critical for mutualist-pathogen transition in Colletotrichum tofieldiae. Nat Commun. 2023;14(1):5288. 37673872 10.1038/s41467-023-40867-w PMC10482981

[103] Magnoli AP, et al. Novel yeast isolated from broilers’ feedstuff, gut and faeces as aflatoxin B1 adsorbents. J Appl Microbiol. 2016;121(6):1766–76. 27638385 10.1111/jam.13297

[104] Itani K, et al. Effect of Cyberlindnera jadinii yeast on growth performance, nutrient digestibility, and gut health of broiler chickens from 1 to 34 d of age. Poult Sci. 2023;102(12):103127. 37837676 10.1016/j.psj.2023.103127 PMC10585334

[105] Angulo M, et al. Probiotic and nutritional effects of Debaryomyces hansenii on animals. Appl Microbiol Biotechnol. 2020;104(18):7689–99. 32686006 10.1007/s00253-020-10780-z

[106] Gonzalez-Dominguez E, et al. Development and validation of a mechanistic model that predicts infection by Diaporthe ampelina, the causal agent of Phomopsis cane and leaf spot of grapevines. Front Plant Sci. 2022;13:872333. 35463401 10.3389/fpls.2022.872333 PMC9021785

[107] Yerlikaya O, Akan E, Kinik Ö. The metagenomic composition of water kefir microbiota. International Journal of Gastronomy and Food Science. 2022;30:100621.

[108] Munkvold GP. Fusarium Species and Their Associated Mycotoxins. Methods Mol Biol. 2017;1542:51–106. 10.1007/978-1-4939-6707-0_4 27924531

[109] Fernández-Pacheco P, et al. Saccharomyces cerevisiae and Hanseniaspora osmophila strains as yeast active cultures for potential probiotic applications. Food Funct. 2019;10(8):4924–31. 31342038 10.1039/c9fo00732f

[110] Jeong DM, et al. Genomic and functional features of yeast species in Korean traditional fermented alcoholic beverage and soybean products. FEMS Yeast Res. 2022;23:foac066. 10.1093/femsyr/foac066 36564017

[111] Homayouni-Rad A, et al. Kluyveromyces marxianus as a probiotic yeast: a mini-review. Curr Nutr Food Sci. 2020;16(8):1163–9.

[112] Agarbati A, et al. Potential Probiotic Yeasts Sourced from Natural Environmental and Spontaneous Processed Foods. Foods. 2020;9(3):287. 32143376 10.3390/foods9030287 PMC7143343

[113] Morand S, et al. Complete genome sequence of Malassezia restricta CBS 7877, an opportunist pathogen involved in dandruff and seborrheic dermatitis. Microbiology Resource Announcements. 2019;8:8. 10.1128/MRA.01543-18 PMC6368656 30746521

[114] Wang P, et al. Monascus purpureus M-32 improves growth performance, immune response, intestinal morphology, microbiota and disease resistance in Litopenaeus vannamei. Aquaculture. 2021;530:735947.

[115] Lee Soo C, et al. Analysis of a food-borne fungal pathogen outbreak: virulence and genome of a mucor circinelloides isolate from yogurt. mBio. 2014;5(4). 10.1128/mbio.01390-14. 10.1128/mBio.01390-14 PMC4161253 25006230

[116] Chávez R, et al. Mold-fermented foods: Penicillium spp. as ripening agents in the elaboration of cheese and meat products. Mycofactories. 2011;26:73–98.

[117] Geltner C, et al. Invasive pulmonary mycosis due to penicillium chrysogenum: a new invasive pathogen. Transplantation. 2013;95(4):e21. 23423272 10.1097/TP.0b013e31827ff214

[118] Frisvad JC. 10 - Rationale for a polyphasic approach in the identification of mycotoxigenic fungi, in determining Mycotoxins and Mycotoxigenic Fungi in Food and Feed. S. De Saeger, editor. Cambridge: Woodhead Publishing; 2011. p. 279–297.

[119] Faid M, Tantaoui-Elaraki A. Production of toxic metabolites by Penicillium italicum and P. digitatum Isolated from Citrus Fruits. J Food Prot. 1989;52(3):194–7. 30991508 10.4315/0362-028X-52.3.194

[120] Sawant AM, et al. Morphological and molecular characterization of Penicillium rubens sp.nov isolated from poultry feed. Indian Phytopathology. 2019;72(3):461–78.

[121] Lv X, et al. Endophytic fungus Penicillium steckii DF33 promoted tanshinones biosynthesis in Salvia miltiorrhiza by regulating the expression of CYP450 genes. Gene. 2024;899:148094. 38142897 10.1016/j.gene.2023.148094

[122] Ismaiel AA, Papenbrock J. The effects of patulin from Penicillium vulpinum on seedling growth, root tip ultrastructure and glutathione content of maize. Eur J Plant Pathol. 2014;139(3):497–509.

[123] Sprute R, et al. Invasive infections with Purpureocillium lilacinum: clinical characteristics and outcome of 101 cases from FungiScope® and the literature. J Antimicrob Chemother. 2021;76(6):1593–603. 33599275 10.1093/jac/dkab039 PMC8120338

[124] Fujimoto A, et al. The first case of cutaneous mucormycosis caused by Rhizopus azygosporus. Br J Dermatol. 2005;153(2):428–30. 16086761 10.1111/j.1365-2133.2005.06593.x

[125] Corzo-León DE, Uehling JK, Ballou ER. Microbe of the month: Rhizopus arrhizus. Trends Microbiol. 2023;31(9):985–7. 10.1016/j.tim.2023.03.013 37062623

[126] Cheng Vincent CC, et al. Outbreak of intestinal infection due to Rhizopus microsporus. J Clin Microbiol. 2009;47(9):2834–43. 19641069 10.1128/JCM.00908-09 PMC2738128

[127] Hof H. Rhodotorula spp. in the gut - foe or friend? GMS Infect Dis. 2019;7:Doc02. 31538040 10.3205/id000042 PMC6734584

[128] Shareef A, Al-Dabbagh A. Effect of probiotic (Saccharomyces cerevisiae) on performance of broiler chicks. Iraqi J Vet Med. 2009;23:23–9.

[129] Hittinger CT, Steele JL, Ryder DS. Diverse yeasts for diverse fermented beverages and foods. Curr Opin Biotechnol. 2018;49:199–206. 29102814 10.1016/j.copbio.2017.10.004

[130] Wang F, Han R, Chen S. An overlooked and underrated endemic mycosis—talaromycosis and the pathogenic fungus Talaromyces marneffei. Clin Microbiol Rev. 2023;36(1):e00051-e00122. 36648228 10.1128/cmr.00051-22 PMC10035316

[131] Wang L, et al. Fruit rot on tomatoes caused by Talaromyces stipitatus newly reported in China. Crop Prot. 2024;181:106698.

[132] Helmy EA, et al. Does Torulaspora delbrueckii has some probiotic capabilities? In vitro and in vivo assessment. Nutrire. 2024;49(1):15.

[133] Gezgin Y, et al. Evaluation of Trichoderma atroviride and Trichoderma citrinoviride growth profiles and their potentials as biocontrol agent and biofertilizer. Turkish Journal of Biochemistry. 2020;45(2):163–75.

[134] García-Hernández Y, et al. Identification and in vitro screening of avian yeasts for use as probiotic. Res Vet Sci. 2012;93(2):798–802. 22047814 10.1016/j.rvsc.2011.09.005

[135] Fuloria S, et al. Synbiotic effects of fermented rice on human health and wellness: a natural beverage that boosts immunity. Front Microbiol. 2022;13:950913. 35910609 10.3389/fmicb.2022.950913 PMC9325588

[136] McCafferty KW, et al. Effects of age and supplemental xylanase in corn- and wheat-based diets on cecal volatile fatty acid concentrations of broilers1. Poult Sci. 2019;98(10):4787–800. 31065717 10.3382/ps/pez194

[137] Leung H, Kiarie EG. Standardized ileal digestibility of amino acids and apparent metabolizable energy in corn and soybean meal for organic broiler chicken production in Ontario. Can J Anim Sci. 2020;100(3):447–54.

[138] Jafari Arvari AR, et al. A comparative study on the effect of limestone particle size on performance, ileal digestibility of calcium and phosphorus, and bone characteristics in broilers and pullets. Br Poult Sci. 2024;65(1):52–61. 37861101 10.1080/00071668.2023.2272966

[139] Siegert W, et al. Amino acid digestibility and metabolizable energy of soybean meal of different origins in cecectomized laying hens. Poult Sci. 2023;102(5):102580. 36913760 10.1016/j.psj.2023.102580 PMC10024217

[140] Bansal M, et al. A secondary bile acid from microbiota metabolism attenuates ileitis and bile acid reduction in subclinical necrotic enteritis in chickens. Journal of Animal Science and Biotechnology. 2020;11(1):37. 32190299 10.1186/s40104-020-00441-6 PMC7069026

[141] Loy DD, EL Lundy. Chapter 23 - Nutritional properties and feeding value of corn and its coproducts. In: Serna-Saldivar SO, editor. Corn (Third Edition). AACC International Press: Oxford; 2019. p. 633–59.

[142] Zhou Q, et al. The spatial and temporal characterization of gut microbiota in broilers. Front Vet Sci. 2021;8:712226. 34527716 10.3389/fvets.2021.712226 PMC8435590

[143] Rohatgi A. WebPlotDigitizer. 2024. Available from: https://automeris.io.

[144] Foundation, P.S., Python 3.8. 2019. Available from: https://www.python.org.

[145] The pandas development team. Pandas-dev/pandas: Pandas. Zenodo; 2020. Available from: 10.5281/zenodo.3509134.

[146] Hunter JD. Matplotlib: a 2D graphics environment. Computing in Science & Engineering. 2007;9(3):90–5.

[147] Waskom ML. Seaborn: statistical data visualization. Journal of Open Source Software. 2021;6(60):3021.

[148] Achiam J, Development Team. Gpt-4 technical report. 2023. arXiv preprint arXiv:2303.08774.

[149] Gómez-Cerezo J, et al. Achromobacter xylosoxidans bacteremia: a 10-year analysis of 54 cases. Eur J Clin Microbiol Infect Dis. 2003;22:360–3. 12750959 10.1007/s10096-003-0925-3

[150] Afridi OK, Ali J, Chang JH. Next-Generation Sequencing Based Gut Resistome Profiling of Broiler Chickens Infected with Multidrug-Resistant Escherichia coli. Animals. 2020;10(12):2350. 10.3390/ani10122350 PMC7764233 33317082

[151] Zhou Y, et al. Fine particulate matter perturbs the pulmonary microbiota in broiler chickens. Animals. 2023;13(18):2862. 37760262 10.3390/ani13182862 PMC10525718

[152] Yan L, et al. Effects of corn particle size on growth performance, gastrointestinal development, carcass indices and intestinal microbiota of broilers. Poult Sci. 2022;101(12):102205. 36370669 10.1016/j.psj.2022.102205 PMC9664518

[153] Wang T, et al. Acinetobacter radioresistens infection with bacteremia and pneumonia. IDCases. 2019;15:e00495. 30906692 10.1016/j.idcr.2019.e00495 PMC6411504

[154] Khafagy, A, ELTarabili, R, Yousseff, F, Abo Hashem, M, El Attar, Y. Importance of Aerococcus viridans in diarrhea in poultry in Egypt. Suez Canal Vet Med J. 2023. 10.21608/SCVMJ.2023.195149.1121.

[155] Biben I, et al. Potentiation of probiotic activity by simultaneous use of aerococcus viridans and mycobacterium vaccae. Scientific and Technical Bulletin of State Scientific Research Control Institute of Veterinary Medical Products and Fodder Additives and Institute of Animal Biology. 2023;24(1):18–26.

[156] Igbinosa IH. Antibiogram profiling and pathogenic status of Aeromonas species recovered from Chicken. Saudi J Biol Sci. 2014;21(5):481–5. 25313284 10.1016/j.sjbs.2014.06.003 PMC4190985

[157] Chaves B, Brashears M, Nightingale K. Applications and safety considerations of Lactobacillus salivarius as a probiotic in animal and human health. J Appl Microbiol. 2017;123(1):18–28. 28256040 10.1111/jam.13438

[158] Yang W-Y, Chou C-H, Wang C. The effects of feed supplementing Akkemansia muciniphila on incidence, severity, and gut microbiota of necrotic enteritis in chickens. Poult Sci. 2022;101(4):101751. 35240353 10.1016/j.psj.2022.101751 PMC8889413

[159] Farkas V, et al. Microbiota composition of mucosa and interactions between the microbes of the different gut segments could be a factor to modulate the growth rate of broiler chickens. Animals. 2022;12(10):1296. 35625142 10.3390/ani12101296 PMC9137591

[160] Sun B, Hou L, Yang Y. The development of the gut microbiota and short-chain fatty acids of layer chickens in different growth periods. Frontiers in Veterinary Science. 2021;8:666535. 34277754 10.3389/fvets.2021.666535 PMC8284478

[161] Fan Y, et al. Week-old chicks with high Bacteroides abundance have increased short-chain fatty acids and reduced markers of gut inflammation. Microbiology Spectrum. 2023;11(2):e03616–22. 36719194 10.1128/spectrum.03616-22 PMC10100795

[162] El-Moneim AEMEA, et al. Assessment of in ovo administration of Bifidobacterium bifidum and Bifidobacterium longum on performance, ileal histomorphometry, blood hematological, and biochemical parameters of broilers. Probiotics Antimicrob Proteins. 2020;12:439–50. 31025259 10.1007/s12602-019-09549-2

[163] Liu X, et al. Blautia—a new functional genus with potential probiotic properties? Gut microbes. 2021;13(1):1875796. 33525961 10.1080/19490976.2021.1875796 PMC7872077

[164] Purba MA, et al. A study about protective effect of Brevibacillus laterosporus texasporus culture on broiler chickens infected with Salmonella pullorum. International Journal of Science, Technology & Management. 2020;1(2):68–78.

[165] Eeckhaut V, et al. The probiotic Butyricicoccus pullicaecorum reduces feed conversion and protects from potentially harmful intestinal microorganisms and necrotic enteritis in broilers. Front Microbiol. 2016;7:218034. 10.3389/fmicb.2016.01416 PMC5030265 27708624

[166] Fukuda S, et al. Isolation of a novel strain of Butyrivibrio fibrisolvens that isomerizes linoleic acid to conjugated linoleic acid without hydrogenation, and its utilization as a probiotic for animals. J Appl Microbiol. 2006;100(4):787–94. 16553734 10.1111/j.1365-2672.2006.02864.x

[167] Silva J, et al. Campylobacter spp. as a foodborne pathogen: a review. Front Microbiol. 2011;2:12125. 10.3389/fmicb.2011.00200 PMC3180643 21991264

[168] Sharma A, Gilbert JA, Lal R. (Meta) genomic insights into the pathogenome of Cellulosimicrobium cellulans. Sci Rep. 2016;6(1):25527. 27151933 10.1038/srep25527 PMC4858710

[169] Xie M, et al. Growth performance, hepatic enzymes, and gut health status of common carp (Cyprinus carpio) in response to dietary Cetobacterium somerae fermentation product. Aquaculture Reports. 2022;23:101046.

[170] Harvey RB, et al. Clostridium difficile in poultry and poultry meat. Foodborne Pathog Dis. 2011;8(12):1321–3. 21877928 10.1089/fpd.2011.0936

[171] Liu T, et al. Evaluation of dynamic effects of dietary medium-chain monoglycerides on performance, intestinal development and gut microbiota of broilers in large-scale production. Animal Nutrition. 2023;14:269–80. 37600838 10.1016/j.aninu.2023.05.003 PMC10432913

[172] Liu J, et al. Anaerobutyricum and Subdoligranulum are differentially enriched in broilers with disparate weight gains. Animals. 2023;13(11):1834. 37889711 10.3390/ani13111834 PMC10251939

[173] Ryan MP, et al. The emergence of the genus Comamonas as important opportunistic pathogens. Pathogens. 2022;11(9):1032. 36145464 10.3390/pathogens11091032 PMC9504711

[174] Burkovski A. Corynebacterium pseudodiphtheriticum: putative probiotic, opportunistic infector, emerging pathogen. New York: Taylor and Francis; 2015. p. 673–674. 10.1080/21505594.2015.1067747 PMC4720244 26252066

[175] Ruiza D, et al. Characterization and screening of plant probiotic traits of bacteria isolated from rice seeds cultivated in Argentina. The Journal of Microbiology. 2011;49:902–12. 22203552 10.1007/s12275-011-1073-6

[176] Jin YY, et al. Effect of heat stress on ileal microbial community of indigenous yellow-feather broilers based on 16S rRNA gene sequencing. Veterinary Medicine and Science. 2022;8(2):642–53. 35040272 10.1002/vms3.734 PMC8959285

[177] Click RE. Successful treatment of asymptomatic or clinically terminal bovine Mycobacterium avium subspecies paratuberculosis infection (Johne’s disease) with the bacterium Dietzia used as a probiotic alone or in combination with dexamethasone: adaption to chronic human diarrheal diseases. Virulence. 2011;2(2):131–43. 21460639 10.4161/viru.2.2.15647 PMC3265756

[178] Ahn SB, et al. Randomized, double-blind, placebo-controlled study of a multispecies probiotic mixture in nonalcoholic fatty liver disease. Sci Rep. 2019;9(1):5688. 30952918 10.1038/s41598-019-42059-3 PMC6450966

[179] Zhang S, et al. Dietary supplementation with Bacillus subtilis promotes growth performance of broilers by altering the dominant microbial community. Poult Sci. 2021;100(3):100935. 33652528 10.1016/j.psj.2020.12.032 PMC7936199

[180] Guo J, et al. High-throughput sequencing reveals the effect of Bacillus subtilis CGMCC 1.921 on the cecal microbiota and gene expression in ileum mucosa of laying hens. Poult Sci. 2018;97(7):2543–56. 29897524 10.3382/ps/pey112

[181] Wang L, et al. Effect of stocking density on performance, meat quality and cecal bacterial communities of yellow feather broilers. Anim Biotechnol. 2022;33(6):1322–32. 33752552 10.1080/10495398.2021.1898413

[182] Lei J, et al. Intestinal microbiota regulate certain meat quality parameters in chicken. Front Nutr. 2022;9:747705. 35548562 10.3389/fnut.2022.747705 PMC9085416

[183] Allameh SK, et al. Isolation, identification and characterization of Leuconostoc mesenteroides as a new probiotic from intestine of snakehead fish (Channa striatus). Afr J Biotech. 2012;11(16):3810–6.

[184] Yan C, et al. Exogenous fecal microbial transplantation alters fearfulness, intestinal morphology, and gut microbiota in broilers. Frontiers in Veterinary Science. 2021;8:706987. 34660756 10.3389/fvets.2021.706987 PMC8517117

[185] Wan Y, et al. Effects of different-sized cages on the production performance, serum parameters, and caecal microbiota composition of laying hens. Animals. 2023;13(2):266. 36670806 10.3390/ani13020266 PMC9854594

[186] Liu L, et al. Effects of Lactiplantibacillus plantarum LPJZ-658 supplementation on the production, meat quality, intestinal morphology, and cecal microbiota of broilers chickens. Microorganisms. 2023;11(6):1549. 37375050 10.3390/microorganisms11061549 PMC10301381

[187] Ekim B, et al. Effects of Paenibacillus xylanexedens on growth performance, intestinal histomorphology, intestinal microflora, and immune response in broiler chickens challenged with Escherichia coli K88. Poult Sci. 2020;99(1):214–23. 32416805 10.3382/ps/pez460 PMC7587685

[188] Ju T, et al. Defining the role of Parasutterella, a previously uncharacterized member of the core gut microbiota. ISME J. 2019;13(6):1520–34. 30742017 10.1038/s41396-019-0364-5 PMC6776049

[189] Oba PM, et al. Effects of passive immunization by anti-gingipain IgY on the oral health of cats fed kibble diets. J Vet Dent. 2018;35(4):275–80.

[190] Kumar S, Shang Y, Kim WK. Insight into dynamics of gut microbial community of broilers fed with fructooligosaccharides supplemented low calcium and phosphorus diets. Frontiers in veterinary science. 2019;6:95. 30984773 10.3389/fvets.2019.00095 PMC6449842

[191] Li A, et al. Microbiome analysis reveals gut microbiota alteration of early-weaned Yimeng black goats with the effect of milk replacer and age. Microb Cell Fact. 2021;20:1–14. 33789672 10.1186/s12934-021-01568-5 PMC8010993

[192] Lv H, et al. Microbial composition in the duodenum and ileum of yellow broilers with high and low feed efficiency. Front Microbiol. 2021;12:689653. 34385985 10.3389/fmicb.2021.689653 PMC8353196

[193] Bitterncourt LC, et al. Influence of a probiotic on broiler performance. Revista Brasileira de Zootecnia. 2011;40:2739–43.

[194] Baker-Austin C, et al. Vibrio spp. infections. Nat Rev Dis Primers. 2018;4(1):1–19. 30002421 10.1038/s41572-018-0005-8

[195] Davies CP, et al. Temporal dynamics of the chicken mycobiome. Front Physiol. 2022;13:1057810. 36589448 10.3389/fphys.2022.1057810 PMC9799259

[196] Ma C, et al. Differential pattern of indigenous microbiome responses to probiotic Bifidobacterium lactis V9 consumption across subjects. Food Res Int. 2020;136:109496. 32846577 10.1016/j.foodres.2020.109496

[197] Pan D, Yu Z. Intestinal microbiome of poultry and its interaction with host and diet. Gut Microbes. 2014;5(1):108–19. 24256702 10.4161/gmic.26945 PMC4049927

[198] Tachon S, Lee B, Marco ML. Diet alters probiotic Lactobacillus persistence and function in the intestine. Environ Microbiol. 2014;16(9):2915–26. 24118739 10.1111/1462-2920.12297

[199] Liljebjelke K, et al. Vertical and horizontal transmission of salmonella within integrated broiler production system. Foodborne Pathog Dis. 2005;2:90–102. 15992303 10.1089/fpd.2005.2.90

[200] Oakley BB, et al. The chicken gastrointestinal microbiome. FEMS Microbiol Lett. 2014;360(2):100–12. 25263745 10.1111/1574-6968.12608

[201] Oladeinde A, et al. Litter commensal bacteria can limit the horizontal gene transfer of antimicrobial resistance to Salmonella in chickens. Appl Environ Microbiol. 2022;88(9):e02517-e2521. 35416680 10.1128/aem.02517-21 PMC9107613

[202] Wang L, Yu Z. Intestinal microbiota of broiler chickens as affected by litter management regimens. Front Microbiol. 2016;7:191480. 10.3389/fmicb.2016.00593 PMC4870231 27242676

[203] Cressman MD, et al. Interrelations between the microbiotas in the litter and in the intestines of commercial broiler chickens. Appl Environ Microbiol. 2010;76(19):6572–82. 20693454 10.1128/AEM.00180-10 PMC2950482

[204] Zwirzitz B, et al. Temporal dynamics of the cecal and litter microbiome of chickens raised in two separate broiler houses. Front Physiol. 2023;14:1083192. 36935743 10.3389/fphys.2023.1083192 PMC10018173

[205] Józefiak D, Rutkowski A, Martin SA. Carbohydrate fermentation in the avian ceca: a review. Anim Feed Sci Technol. 2004;113(1):1–15.

[206] Wang J, et al. Effects of xylanase in corn- or wheat-based diets on cecal microbiota of broilers. Front Microbiol. 2021;12:757066. 34721363 10.3389/fmicb.2021.757066 PMC8548762

[207] Denayrolles M , Arturo-schaan M, Massias B, Bebin K, Elie AM, Panheleux-Lebastard M, Urdaci MC. Effect of diets with different fibrous contents on broiler gut microflora and short-chain fatty acid (SCFA) production. In: 16th European Symposium on Poultry Nutrition. 2007. p. 269–272.

[208] Wolfe Alan J. The acetate switch. Microbiol Mol Biol Rev. 2005;69(1):12–50. 15755952 10.1128/MMBR.69.1.12-50.2005 PMC1082793

[209] Valgepea K, et al. Systems biology approach reveals that overflow metabolism of acetate in Escherichia coli is triggered by carbon catabolite repression of acetyl-CoA synthetase. BMC Syst Biol. 2010;4(1):166. 21122111 10.1186/1752-0509-4-166 PMC3014970

[210] Pedroso AA, Batal AB, Lee MD. Effect of in ovo administration of an adult-derived microbiota on establishment of the intestinal microbiome in chickens. Am J Vet Res. 2016;77(5):514–26. 27111019 10.2460/ajvr.77.5.514

[211] Roto SM, Kwon YM, Ricke SC. Applications of in ovo technique for the optimal development of the gastrointestinal tract and the potential influence on the establishment of its microbiome in poultry. Frontiers in veterinary science. 2016;3:63. 27583251 10.3389/fvets.2016.00063 PMC4987676

[212] Teague KD, et al. In ovo evaluation of FloraMax®-B11 on Marek’s disease HVT vaccine protective efficacy, hatchability, microbiota composition, morphometric analysis, and Salmonella enteritidis infection in broiler chickens. Poult Sci. 2017;96(7):2074–82. 28160004 10.3382/ps/pew494

[213] Rodrigues DR, et al. Intestinal pioneer colonizers as drivers of ileal microbial composition and diversity of broiler chickens. Front Microbiol. 2020;10:2858. 31998246 10.3389/fmicb.2019.02858 PMC6962117

[214] Huang P, et al. The chicken gut metagenome and the modulatory effects of plant-derived benzylisoquinoline alkaloids. Microbiome. 2018;6(1):211. 30482240 10.1186/s40168-018-0590-5 PMC6260706

[215] Akinyemi FT, et al. Dynamic distribution of gut microbiota during embryonic development in chicken. Poult Sci. 2020;99(10):5079–90. 32988546 10.1016/j.psj.2020.06.016 PMC7598139

[216] Yang M, et al. Dynamic changes in the gut microbial community and function during broiler growth. Microbiol Spectr. 2022;10(4):e0100522. 35950773 10.1128/spectrum.01005-22 PMC9430649

[217] Ott SJ, et al. Reduction in diversity of the colonic mucosa associated bacterial microflora in patients with active inflammatory bowel disease. Gut. 2004;53(5):685–93. 15082587 10.1136/gut.2003.025403 PMC1774050

[218] Thomas S, et al. The host microbiome regulates and maintains human health: a primer and perspective for non-microbiologists. Cancer Res. 2017;77(8):1783–812. 28292977 10.1158/0008-5472.CAN-16-2929 PMC5392374

[219] Li Z, et al. Effects of Lactobacillus acidophilus on gut microbiota composition in broilers challenged with Clostridium perfringens. PLoS ONE. 2017;12(11):e0188634. 29190649 10.1371/journal.pone.0188634 PMC5708699

[220] Alhotan RA. Commercial poultry feed formulation: current status, challenges, and future expectations. Worlds Poult Sci J. 2021;77(2):279–99.

[221] Fukuda S, et al. Bifidobacteria can protect from enteropathogenic infection through production of acetate. Nature. 2011;469(7331):543–7. 21270894 10.1038/nature09646

[222] Marinos G, et al. Metabolic model predictions enable targeted microbiome manipulation through precision prebiotics. Microbiology Spectrum. 2024;12(2):e01144–223. 38230938 10.1128/spectrum.01144-23 PMC10846184

